# Association of Inappropriate Outpatient Pediatric Antibiotic Prescriptions With Adverse Drug Events and Health Care Expenditures

**DOI:** 10.1001/jamanetworkopen.2022.14153

**Published:** 2022-05-26

**Authors:** Anne M. Butler, Derek S. Brown, Michael J. Durkin, John M. Sahrmann, Katelin B. Nickel, Caroline A. O’Neil, Margaret A. Olsen, David Y. Hyun, Rachel M. Zetts, Jason G. Newland

**Affiliations:** 1 Division of Infectious Diseases, John T. Milliken Department of Medicine, Washington University School of Medicine, St Louis, Missouri; 2 Division of Public Health Sciences, Department of Surgery, Washington University School of Medicine, St Louis, Missouri; 3 Brown School, Washington University, St Louis, Missouri; 4 The Pew Charitable Trusts, Washington, DC; 5 Department of Pediatrics, Washington University School of Medicine, St Louis, Missouri

## Abstract

**Importance:**

Nonguideline antibiotic prescribing for the treatment of pediatric infections is common, but the consequences of inappropriate antibiotics are not well described.

**Objective:**

To evaluate the comparative safety and health care expenditures of inappropriate vs appropriate oral antibiotic prescriptions for common outpatient pediatric infections.

**Design, Setting, and Participants:**

This cohort study included children aged 6 months to 17 years diagnosed with a bacterial infection (suppurative otitis media [OM], pharyngitis, sinusitis) or viral infection (influenza, viral upper respiratory infection [URI], bronchiolitis, bronchitis, nonsuppurative OM) as an outpatient from April 1, 2016, to September 30, 2018, in the IBM MarketScan Commercial Database. Data were analyzed from August to November 2021.

**Exposures:**

Inappropriate (ie, non–guideline-recommended) vs appropriate (ie, guideline-recommended) oral antibiotic agents dispensed from an outpatient pharmacy on the date of infection.

**Main Outcomes and Measures:**

Propensity score–weighted Cox proportional hazards models were used to estimate hazards ratios (HRs) and 95% CIs for the association between inappropriate antibiotic prescriptions and adverse drug events. Two-part models were used to calculate 30-day all-cause attributable health care expenditures by infection type. National-level annual attributable expenditures were calculated by scaling attributable expenditures in the study cohort to the national employer-sponsored insurance population.

**Results:**

The cohort included 2 804 245 eligible children (52% male; median [IQR] age, 8 [4-12] years). Overall, 31% to 36% received inappropriate antibiotics for bacterial infections and 4% to 70% for viral infections. Inappropriate antibiotics were associated with increased risk of several adverse drug events, including *Clostridioides difficile* infection and severe allergic reaction among children treated with a nonrecommended antibiotic agent for a bacterial infection (among patients with suppurative OM, *C. difficile *infection: HR, 6.23; 95% CI, 2.24-17.32; allergic reaction: HR, 4.14; 95% CI, 2.48-6.92). Thirty-day attributable health care expenditures were generally higher among children who received inappropriate antibiotics, ranging from $21 to $56 for bacterial infections and from −$96 to $97 for viral infections. National annual attributable expenditure estimates were highest for suppurative OM ($25.3 million), pharyngitis ($21.3 million), and viral URI ($19.1 million).

**Conclusions and Relevance:**

In this cohort study of children with common infections treated in an outpatient setting, inappropriate antibiotic prescriptions were common and associated with increased risks of adverse drug events and higher attributable health care expenditures. These findings highlight the individual- and national-level consequences of inappropriate antibiotic prescribing and further support implementation of outpatient antibiotic stewardship programs.

## Key Points

**Question:**

Do adverse events and health care expenditures differ in children given inappropriate vs appropriate oral antibiotic prescriptions for common outpatient infections?

**Findings:**

In this cohort study of more than 2.8 million children with commercial insurance, inappropriate antibiotics were associated with increased risk of several adverse drug events (eg, *Clostridioides difficile* infection, severe allergic reaction) and generally higher 30-day all-cause attributable expenditures. National annual expenditure estimates associated with inappropriate antibiotic treatment in the pediatric commercially insured population were highest for suppurative otitis media, pharyngitis, and viral upper respiratory infection.

**Meaning:**

Inappropriate antibiotic prescriptions were associated with avoidable adverse drug events and substantial individual- and national-level health care expenditures.

## Teaser

This cohort study evaluates the comparative safety and health care expenditures of inappropriate vs appropriate oral antibiotic prescriptions for common outpatient pediatric infections.

## Introduction

Approximately 29% of outpatient antibiotics prescribed to children in the United States are inappropriate.^1^ These include a large proportion of children inappropriately prescribed any antibiotic agent for a viral infection (eg, 21% of viral upper respiratory infection [URI] diagnoses)^1^ or inappropriately prescribed a non–first-line antibiotic agent for a bacterial infection (eg, 33% of treated otitis media [OM] diagnoses, 40% of treated pharyngitis diagnoses, and 48% of treated sinusitis diagnoses), ie, non–guideline-concordant antibiotic use.^2,3^ Inappropriate antibiotic prescriptions are harmful on a societal level because they propel the spread of antimicrobial resistance^4^ and harmful on an individual level because they are associated with adverse drug events (ADEs), such as allergic reactions (eg, anaphylaxis, skin rash) and microbiome disruption-related conditions (eg, *Clostridioides difficile* infection).^5,6,7^ The clinical management of antibiotic-resistant infections and antibiotic-related ADEs require costly health care use,^4^ much of which is likely avoidable.^6,7^

Despite inappropriate antibiotic prescribing for the treatment of pediatric infections in the outpatient setting,^1,8^ evidence is limited on the risks related to inappropriate antibiotic prescriptions. Additional study is needed in large, infection-specific cohorts to estimate the comparative risk of individual ADEs among recipients of inappropriate vs appropriate antibiotic prescriptions. Furthermore, comprehensive estimates of attributable health care utilization and expenditures associated with inappropriate antibiotic prescriptions for common outpatient conditions are generally unavailable.^9,10^

The objectives of this study were to evaluate the comparative safety and attributable health care expenditures associated with inappropriate outpatient antibiotic prescriptions for several common bacterial and viral infections, in a cohort of children with commercial insurance in the United States. We also sought to estimate the national-level annual attributable expenditures of inappropriate antibiotic prescriptions for the pediatric commercially-insured population.

## Methods

### Data Source

We used the IBM MarketScan Commercial Database (2015-2018), which contains longitudinal, patient-level data on enrollment and adjudicated inpatient and outpatient insurance claims as well as outpatient pharmacy-dispensed medications for individuals with primarily employer-sponsored commercial insurance and their spouses and dependents.^11^ The institutional review board at Washington University School of Medicine deemed this study exempt from human participant review. This study followed the Strengthening the Reporting of Observational Studies in Epidemiology (STROBE) reporting guideline for cohort studies.

### Study Design and Population

We identified children aged 6 months to 17 years diagnosed in an outpatient setting with a common bacterial infection (suppurative OM, pharyngitis, sinusitis) or viral infection (influenza, viral URI, bronchiolitis [age 6 months to 3 years], bronchitis [age 5 to 17 years], nonsuppurative OM) from April 1, 2016, to September 30, 2018. We constructed cohorts for each infection type based on categories developed by Fleming-Dutra et al^1^; we adapted definitions from the *International Classification of Diseases, Ninth Revision, Clinical Modification* (ICD-9-CM) diagnosis codes to *ICD-10-CM* codes per the Centers for Medicare & Medicaid Services general equivalence mappings^12^ (eTable 1 in the Supplement). The index date was defined as the date of diagnosis (ignoring diagnostic and/or rule-out claims).

Children were required to have continuous health insurance enrollment and prescription drug coverage during the 180-day baseline period before the index date. To restrict the study population to otherwise healthy children with minimal antibiotic exposure, we excluded index events with inpatient or skilled nursing facility admission within 90 days before index, hospice care or mechanical ventilation within 180 days before index, serious underlying medical conditions within 180 days before index (eTables 2 and 3 in the Supplement), a previous diagnosis for the condition of interest within 180 days prior to the index date (eg, a diagnosis that was not eligible as an index event due to other inclusion and exclusion criteria), or antibiotic use (intravenous, intramuscular, oral) within 90 days before index (eTable 2 in the Supplement). We excluded index events with multiple oral antibiotic prescription dispensings or unusual treatment durations (ie, <5 days or >14 days) at index. We applied a tiered approach to study inclusion and exclusion for index events with multiple, simultaneous infection-related diagnoses of interest (eTable 4 in the Supplement).^13^ For study inclusion for viral infection index events, we allowed multiple index diagnoses for which antibiotics are not warranted (ie, other viral index infection). For bacterial infection index events, we allowed multiple diagnoses for which antibiotics are not warranted (ie, index viral infections) and allowed other bacterial index infections (eg, index suppurative OM and sinusitis, assuming patients received first-line antibiotics). For study exclusion, we excluded index events with other diagnoses for which antibiotics are warranted (eTable 4 in the Supplement), irrespective of documentation for a dispensed antibiotic prescription. For example, we excluded bacterial and viral infection index events on the same day as any condition in eTable 4 in the Supplement regardless of antibiotic prescriptions (eg, sinusitis index event also coded for sepsis). We excluded viral infection index events simultaneously coded for a bacterial infection index condition (ie, suppurative OM, sinusitis, or pharyngitis). For nonsuppurative OM, we excluded index events with an antibiotic eardrop prescription at index for expenditure analyses (eTable 2 in the Supplement). Finally, we restricted the population to the first qualifying event per diagnosis per child (eFigure 1 in the Supplement).

### Antibiotic Exposure

An oral antibiotic prescription was linked to an outpatient infection if it occurred on the day of the index diagnosis. We defined 36 index oral antibiotics based on the 2016 antibiotic utilization quality measure in the Healthcare Effectiveness Data and Information Set (eTable 5 in the Supplement).^14^ For bacterial infections, we categorized antibiotic prescriptions by agent as appropriate (ie, first-line antibiotic agent) or inappropriate (ie, non–first-line antibiotic agent) based on treatment guidelines. First-line antibiotic agents included amoxicillin for suppurative OM^15^; amoxicillin or penicillin for pharyngitis^16^; and amoxicillin or amoxicillin-clavulanate for sinusitis.^17^ For viral infections, we categorized antibiotic prescriptions as appropriate (ie, no antibiotic prescription) or inappropriate (ie, antibiotic prescription). Primary analyses focused on bacterial infections because of the use of an active comparator (ie, all children prescribed an antibiotic), which reduces measured and unmeasured confounding in observational studies.^18,19,20^ Secondary analyses focused on viral infections.

### Safety Outcomes

We identified individual ADEs using *ICD-10-CM* diagnosis codes on all medical claims during follow-up (eTable 6 in the Supplement).^21,22,23^ The duration of outcome-specific follow-up periods ranged from 2 to 90 days. To ensure identification of new-onset outcomes, we excluded children diagnosed with the outcome of interest within 30 days prior to the index for each respective ADE. Analyses for the *C. difficile* outcome were restricted to children aged 2 to 17 years.

### Health Care Expenditure Outcomes

Health care expenditures were computed as the sum of out-of-pocket patient expenditures (copayments, coinsurance, deductible) and health plan expenditures (negotiated fees paid to providers [defined in the data source as individual clinicians and facilities] for services including coordination of benefits). We used 2 outcome definitions to compute 30-day expenditures recorded on medical and pharmacy claims: (1) all-cause health care expenditures represented an upper bound by including expenditures recorded on all claims and (2) ADE-associated health care expenditures represented a lower bound by only including expenditures recorded on claims with antibiotic-related ADEs of interest. We included all claims billed with diagnosis codes for select ADEs, provided that the initial ADE-related code occurred within the specified follow-up window. We examined total expenditures and expenditures by setting (inpatient medical, emergency department medical, outpatient medical, outpatient pharmacy). Expenditures were inflation adjusted to 2018 US dollars using the medical care component of the Consumer Price Index.^24^

### Covariates

Baseline covariates were assessed during a 180-day baseline period before the index antibiotic prescription. Potential confounders of the association between antibiotic exposure and ADE outcomes were identified a priori based on clinical knowledge, and included age, sex, health insurance plan type, urban vs rural residence, geographic region, month and year of index, provider specialty, provider location, number of emergency department encounters, and number of unique medication therapeutic groups.^25^ Additional potential confounders incorporated into the expenditure analyses included mean monthly medical and prescription expenditures, number of office visits, frailty markers (eTable 7 in the Supplement), and comorbid conditions defined using the Elixhauser classification (eTable 8 in the Supplement).^26,27^

### Statistical Analysis

Data analyses were performed from August to November 2021. We used stabilized inverse probability of treatment (IPT) weights to balance treatment groups within each cohort with respect to potential confounding factors. We used logistic regression to estimate the propensity of appropriate (vs inappropriate) antibiotic agent, conditional on baseline covariates. Propensity scores were used to create weighted cohorts to estimate the treatment effects in the total population, ie, the average treatment effect (eMethods in the Supplement).^28,29^ We assessed the balance of observed covariates between treatment groups; absolute standardized mean differences of less than 10% in the weighted population were considered adequate.^30^

To examine the association between inappropriate antibiotic agents and each ADE outcome, we used Cox proportional hazards models to estimate unadjusted and weighted hazard ratios (HRs). We used robust variance estimators to calculate 95% CIs.^31^ Children were censored at the end of the outcome-specific follow-up period, end of continuous coverage, subsequent antibiotic prescription for a different agent (eTable 5 in the Supplement), hospitalization, or end of study (December 31, 2018). We selected tendinopathy (including tendon rupture) as a negative control outcome because it is known to be causally unrelated to the exposure (ie, nonfluoroquinolone antibiotics commonly prescribed to children to treat the infections of interest). Although fluoroquinolone antibiotics are associated with tendinopathy,^32^ fluoroquinolones are rarely prescribed to treat the pediatric conditions under study. Given the absence of a biologically plausible mechanism for nonfluoroquinolone antibiotics to cause tendinopathy, estimates of tendinopathy should be null in the absence of confounding.^33^

To estimate attributable expenditures, we used 2-part models. Part 1 was a logistic regression of any vs no expenditures, and part 2 was a flexible model of the level of health care expenditures from a generalized linear model with a log-link and gamma distribution.^34,35^ The modified Park test was used to guide selection of the appropriate distribution.^36,37^ The attributable expenditure was then estimated as the marginal effect (in dollars) that combines both parts. We computed 95% CIs using a nonparametric bootstrap based on 250 resamples.^38,39^ These analyses were restricted to children with continuous health insurance coverage for 30 days of follow-up after index.

To estimate the financial burden of inappropriate antibiotic prescriptions on the US health care system, we scaled the attributable expenditure estimates in the study cohort to the national employer-sponsored insurance population. We standardized and scaled all index events in 2017 to the national employer-sponsored insurance population using MarketScan weights constructed from the American Community Survey with respect to census division, age group, sex, and relationship to the insurance policy holder. We used the calculated IPT-weighted all-cause attributable expenditures to estimate total national-level expenditures for inappropriate antibiotics.

We performed a priori analyses for asthma and allergy, a noninfectious clinical condition frequently treated contrary to guidelines with antibiotic prescriptions, and a subset analyses for asthma exacerbation, applying study inclusion and exclusion criteria as per viral infections. For the all-cause expenditure analyses, we (1) redefined inappropriate antibiotic exposure as inappropriate agent or duration for bacterial infections (eMethods in the Supplement); (2) extended follow-up to 90 days; and (3) excluded beneficiaries with health maintenance organization and point of service with capitation plans.

Analysis was conducted with SAS version 9.4 (SAS Institute). Statistical significance was defined as the absence of the null value within the 95% CIs.

## Results

The study sample included 1 601 019 bacterial infection index events (601 711 [38%] suppurative OM, 617 215 [39%] pharyngitis, and 382 093 [24%] sinusitis) and 1 203 226 viral infection index events (180 996 [15%] influenza, 772 040 [64%] viral URI, 23 931 [2%] bronchiolitis, 72 407 [6%] bronchitis, and 153 852 [13%] nonsuppurative OM) (eFigure 1 in the Supplement). The study sample had a median (IQR) age of 8 (4-12) years, 52% were male, and 48% resided in the South. The proportion of children who received inappropriate antibiotics differed by cohort (bacterial infections: sinusitis, 137 065 [36%]; pharyngitis, 208 705 [34%]; and suppurative OM, 186 832 [31%]; viral infections: bronchitis, 50 806 [70%], nonsuppurative OM, 73 368 [48%]; viral URI, 93 013 [12%]; bronchiolitis, 2120 [9%]; and influenza, 6817 [4%]) (Table 1). The distribution of antibiotic agents differed by infection type (eTable 9 in the Supplement). For example, children with pharyngitis were inappropriately treated with azithromycin (13%), cefdinir (8%), amoxicillin-clavulanate (6%), cephalexin (5%), and other agents (2%). Table 1 and eTable 10 in the Supplement summarize baseline characteristics by exposure group.

**Table 1.** Selected Baseline Characteristics of Infections of Interest Among Children^a^

Characteristic	Participants, No. (%) (N = 2 804 245)			
	Bacterial infections (primary analysis)^b^		Viral infections (secondary analysis)^c^	
	Appropriate antibiotic (n = 1 068 417)	Inappropriate antibiotic (n = 532 602)	Appropriate antibiotic (n = 977 102)	Inappropriate antibiotic (n = 226 124)
**Demographic characteristics**				
Age, mean (SD), y^d^	7 (5)	9 (5)	7 (5)	8 (5)
Sex				
Male	531 360 (49.7)	267 618 (50.3)	505 091 (51.8)	115 988 (51.3)
Female	537 057 (50.3)	264 984 (49.8)	472 011 (48.3)	110 136 (48.7)
Urban residence	860 296 (80.5)	405 581 (76.2)	801 688 (82.1)	175 341 (77.5)
Geographic region				
Midwest	239 621 (22.4)	99 628 (18.7)	182 162 (18.6)	47 279 (20.9)
Northeast	192 453 (18.0)	79 496 (14.9)	183 343 (18.8)	34 893 (15.4)
South	498 727 (46.7)	295 115 (55.4)	449 866 (46.0)	112 327 (49.7)
West	137 616 (12.9)	58 363 (11.0)	161 731 (16.6)	31 625 (14.0)
Health insurance plan type				
Basic, comprehensive	155 208 (14.5)	68 225 (12.8)	141 964 (14.5)	30 539 (13.5)
CDHP	146 332 (13.7)	72 333 (13.6)	125 526 (12.9)	30 391 (13.4)
EPO or PPO	586 972 (54.9)	302 112 (56.7)	533 125 (54.6)	127 185 (56.3)
HMO	95 896 (9.0)	42 401 (8.0)	91 203 (9.3)	18 511 (8.2)
POS or POS with capitation	65 335 (6.1)	38 486 (7.2)	64 807 (6.6)	15 410 (6.8)
Unknown	18 674 (1.8)	9045 (1.7)	20 477 (2.1)	4088 (1.8)
**Index characteristics**				
Index diagnosis				
Suppurative OM	414 879 (38.8)	186 832 (35.1)	NA	NA
Pharyngitis	408 510 (38.2)	208 705 (39.2)	NA	NA
Sinusitis	245 028 (22.9)	137 065 (25.7)	NA	NA
Influenza	NA	NA	174 179 (17.8)	6817 (3.0)
Viral URI	NA	NA	679 027 (69.5)	93 013 (41.1)
Bronchiolitis	NA	NA	21 811 (2.2)	2120 (0.9)
Bronchitis	NA	NA	21 601 (2.2)	50 806 (22.5)
Nonsuppurative OM	NA	NA	80 484 (8.2)	73 368 (32.5)
Index provider specialty				
Emergency medicine	22 312 (2.1)	11 891 (2.2)	36 677 (3.8)	5891 (2.6)
Internal medicine	23 261 (2.2)	15 911 (3.0)	20 679 (2.1)	7587 (3.4)
Other or unknown	304 565 (28.5)	14 6359 (27.5)	26 7952 (27.4)	64 306 (28.4)
Pediatrics or family medicine	718 279 (67.2)	358 441 (67.3)	651 794 (66.7)	148 340 (65.6)
Index provider location				
Emergency department	5748 (0.5)	3087 (0.6)	42 252 (4.3)	1884 (0.8)
Office	947 102 (88.7)	474 315 (89.1)	844 585 (86.4)	196 686 (87.0
Other or unknown	17 713 (1.7)	7856 (1.5)	20 411 (2.1)	4029 (1.8
Retail clinic	3649 (0.3)	1145 (0.2)	1869 (0.2)	414 (0.2
Urgent care center	94 205 (8.8)	46 199 (8.7)	67 985 (7.0)	23 111 (10.2)
**Prior health care utilization**				
Prior emergency department visit	73 220 (6.9)	36 947 (6.9)	61 648 (6.3)	14 019 (6.2)
Prior No. of office visits, median (IQR)	1 (0-3)	1 (0-3)	1 (0-2)	1 (0-2)
Prior No. of unique medication classes, median (IQR)	0 (0-1)	1 (0-1)	0 (0-1)	0 (0-1)

Abbreviations: CDHP, consumer directed health plans; EPO, exclusive provider organization; HMO, health maintenance organization; NA, not applicable; OM, otitis media; POS, point-of-service; PPO, preferred provider organization; URI, upper respiratory infection.

^a^ Demographic and index characteristics were assessed on the index date. Prior health care utilization was assessed in the 180-day baseline period before the index date (days −180 to −1).

^b^ For children diagnosed with bacterial infections (ie, suppurative OM, pharyngitis, or sinusitis), antibiotic prescriptions were categorized as appropriate (ie, first-line antibiotic agent) or inappropriate (ie, non-first-line antibiotic agent); patients without an antibiotic prescription were excluded. First-line antibiotic agents were defined as amoxicillin for suppurative OM; amoxicillin or penicillin for pharyngitis; and amoxicillin or amoxicillin-clavulanate for sinusitis.

^c^ For children diagnosed with viral infections (ie, influenza, viral URI, bronchiolitis, bronchitis, or non-suppurative OM), antibiotic prescriptions were categorized as appropriate (no antibiotic) or inappropriate (antibiotic).

^d^ Bronchiolitis cohort was restricted to ages 6 months to 3 years; bronchitis cohort was restricted to ages 5 to 17 years.

### ADEs

**Figure 1.**
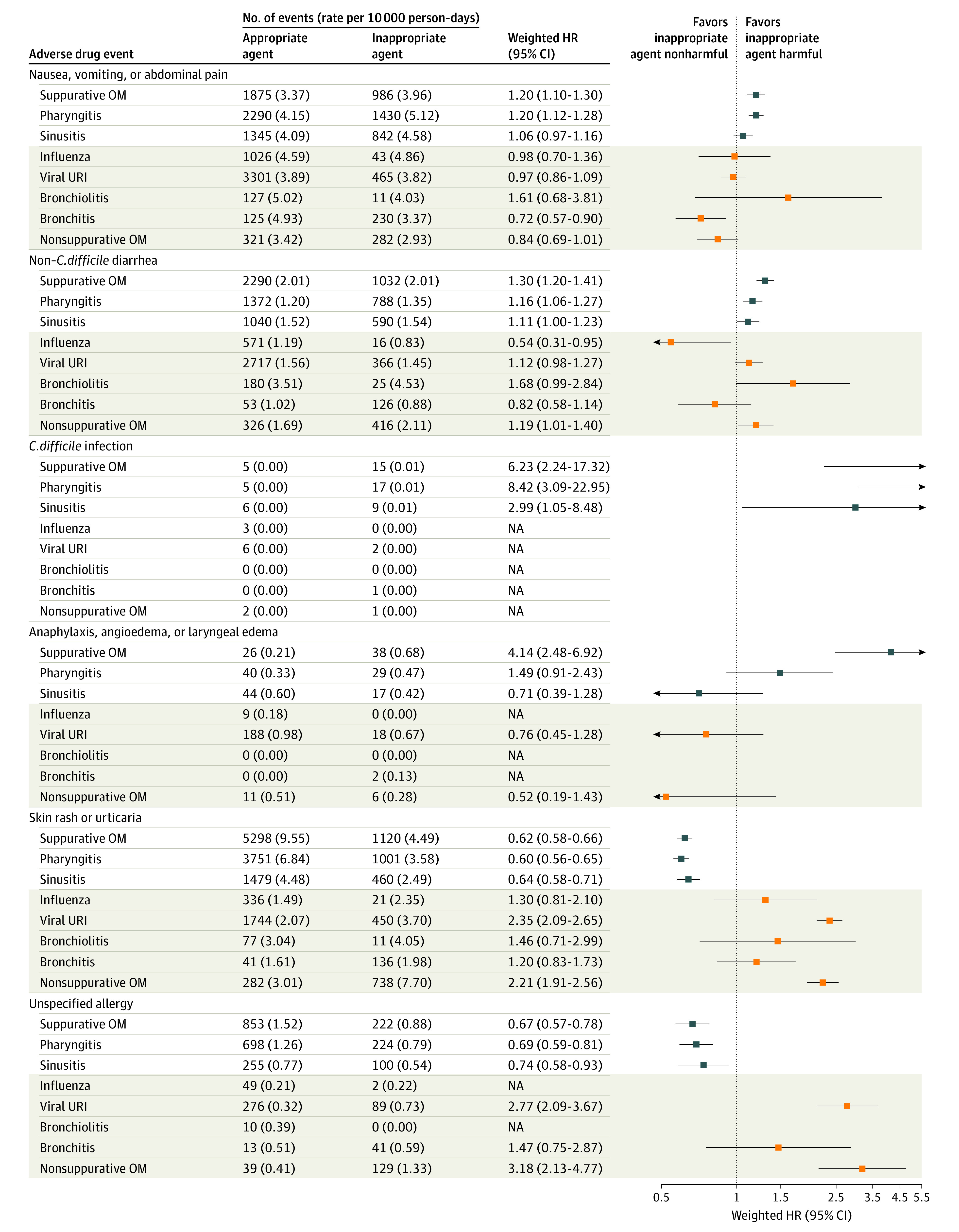
Hazard Ratio (HR) Estimates of Adverse Drug Events Following Inappropriate vs Appropriate Antibiotic Prescriptions Among Pediatric Patients Between 0.0% and 1.8% patients were excluded for 30-day safety outcomes (eTable 11 in the Supplement). Definitions of appropriate and inappropriate agents for bacterial and viral infections are provided in the Methods section. For HR estimation, at least 5 adverse event cases were required in both the reference category (ie, appropriate antibiotic prescription) and the comparator group (ie, inappropriate antibiotic prescription) to ensure stability of the effect estimate. Results for bacterial infections are denoted by a white background with blue boxes; viral infections, brown background with orange boxes. OM indicates otitis media; URI, upper respiratory infection.

After propensity score weighting and outcome-specific exclusions (eTable 11 in the Supplement), exposure groups were similar with respect to baseline characteristics, except for provider specialty and month of index in some cohorts (eFigure 2 in the Supplement). For each infection-specific cohort, case counts, rates, and unadjusted and weighted HR estimates of each ADE outcome following appropriate vs inappropriate antibiotic prescriptions are presented in Figure 1 and eFigure 3 and eTable 12 in the Supplement. Rates of adverse events varied widely, ranging from 0.00 to 0.01 cases per 10 000 person-days for Stevens-Johnson syndrome or toxic epidermal necrolysis to 1.49 to 9.55 cases per 10 000 person-days for skin rash or urticaria.

For children with bacterial infections, inappropriate antibiotic prescriptions were usually associated with higher risk of *C. difficile* infection (eg, children with suppurative OM: HR, 6.23; 95% CI, 2.24-17.32); non–*C. difficile* diarrhea (eg, children with suppurative OM: HR, 1.30; 95% CI, 1.20-1.41); and nausea, vomiting, or abdominal pain (eg, children with suppurative OM: HR, 1.20; 95% CI, 1.10-1.30) and lower risk of skin rash or urticaria (eg, children with suppurative OM: HR, 0.62; 95% CI, 0.58-0.66) as well as unspecified allergy (eg, children with suppurative OM: HR, 0.67; 95% CI, 0.57-0.78) (Figure 1). For children with viral infections, inappropriate antibiotic prescriptions were associated with higher risk of skin rash or urticaria as well as unspecified allergy for viral URI and nonsuppurative OM (Figure 1). Case counts were too rare to estimate some effects (ie, Stevens-Johnson syndrome or toxic epidermal necrolysis and acute kidney failure) (eFigure 3 in the Supplement). In the negative control outcome analysis, we observed similar risks of tendinopathy among children who received appropriate vs inappropriate antibiotic prescriptions, as indicated by 95% CIs that included the null value of 1, for all infection cohorts except pharyngitis (eFigure 3 in the Supplement).

### Attributable Expenditures and National Burden

**Figure 2.**
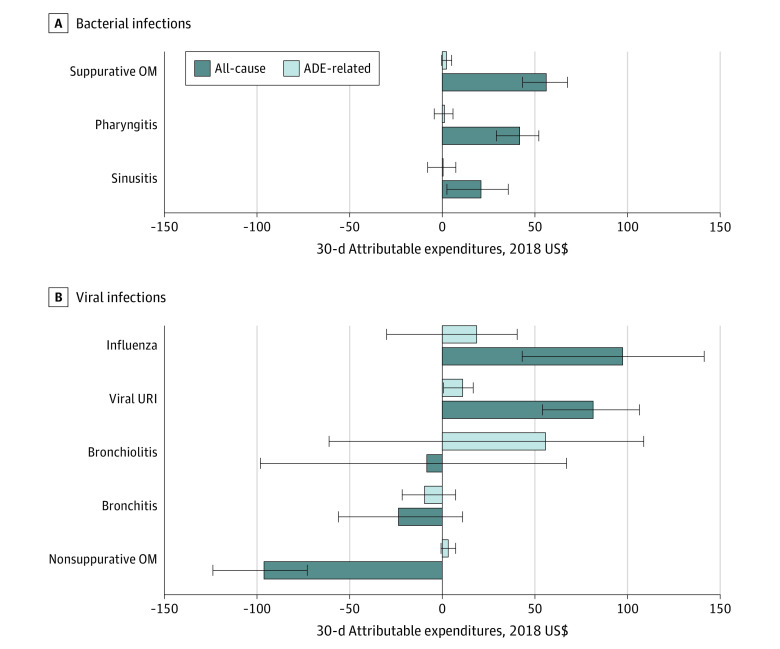
Inverse Probability of Treatment–Weighted 30-Day Patient-Level Attributable Expenditure Estimates of Inappropriate Antibiotic Prescriptions Among Children by Infection Type Black lines indicate 95% CIs. ADE indicates adverse drug event; OM, otitis media; and URI, upper respiratory infection.

After weighting, the exposure groups were similar with respect to baseline characteristics, with few exceptions (eTable 13 in the Supplement). Health care utilization and total per-patient expenditure estimates are presented by infection type for all-cause expenditures (Table 2) and ADE-associated expenditures (eTable 14 in the Supplement). Utilization of inpatient medical care was rare in the 30 days following infection; 0.2% to 0.3% of patients in the bacterial cohort and 0.2% to 1.1% of patients in the viral cohort received inpatient care. For bacterial infections, the mean total attributable expenditure of an inappropriate antibiotic prescription ranged from $21 (95% CI, $3 to $36) for sinusitis to $56 (95% CI, $43 to $68) for suppurative OM; thus, inappropriate vs appropriate antibiotic prescriptions were associated with higher expenditures for suppurative OM, pharyngitis, and sinusitis (Figure 2; eTable 13 in the Supplement). For viral infections, the estimates ranged from −$96 (95% CI, −$124 to −$73) for nonsuppurative OM to $97 (95% CI, $43 to $141) for influenza; thus, inappropriate vs appropriate antibiotic prescriptions were associated with expenditures that were higher for influenza and viral URI, similar for bronchiolitis and bronchitis, and lower for nonsuppurative OM. The ADE-associated attributable expenditure estimates followed a similar pattern but were much closer to the null. The total attributable expenditure differences were largely driven by outpatient pharmacy and outpatient medical utilization and expenditures (eTable 13 in the Supplement).

**Table 2.** Inverse Probability of Treatment–Weighted 30-Day All-Cause Health Care Utilization and Total Per-Patient Expenditure Estimates of Inappropriate Antibiotic Prescriptions Among Children by Setting

Expenditure category	Health care utilization, No. (%)		Total per-patient expenditure estimates, mean (SD), $	
	Appropriate antibiotic	Inappropriate antibiotic	Appropriate antibiotic	Inappropriate antibiotic
**Bacterial infections (primary analysis)**				
Suppurative OM				
Total	402 815 (100.0)	181 486 (100.0)	426 (1985)	498 (2362)
Inpatient medical	954 (0.2)	449 (0.2)	42 (1639)	44 (1869)
Emergency department	10 743 (2.7)	49,12 (2.7)	34 (354)	36 (351)
Outpatient medical	401 128 (99.6)	180 777 (99.6)	300 (815)	315 (988)
Outpatient pharmacy	402 810 (100.0)	181 486 (100.0)	50 (501)	103 (678)
Pharyngitis				
Total	398 702 (100.0)	203 231 (100.0)	392 (2164)	471 (2439)
Inpatient medical	913 (0.2)	477 (0.2)	44 (1837)	50 (2085)
Emergency department	9093 (2.3)	4926 (2.4)	32 (397)	41 (388)
Outpatient medical	397 324 (99.7)	202 460 (99.6)	265 (793)	290 (795)
Outpatient pharmacy	398 697 (100.0)	203 230 (100.0)	51 (539)	90 (639)
Sinusitis				
Total	238 337 (100.0)	133 133 (100.0)	476 (2409)	507 (2684)
Inpatient medical	584 (0.2)	335 (0.3)	53 (1975)	49 (2013)
Emergency department	5217 (2.2)	2956 (2.2)	38 (392)	39 (429)
Outpatient medical	237 976 (99.8)	132 917 (99.8)	307 (997)	311 (1372)
Outpatient pharmacy	238 330 (100.0)	133 131 (100.0)	78 (669)	109 (704)
**Viral infections (secondary analysis)**				
Influenza				
Total	162 779 (100.0)	6366 (100.0)	549 (2254)	644 (1935)
Inpatient medical	382 (0.2)	11 (0.2)	43 (1989)	43 (1537)
Emergency department	8237 (5.1)	324 (5.1)	78 (492)	47 (411)
Outpatient medical	159 233 (97.8)	6201 (97.4)	270 (639)	341 (715)
Outpatient pharmacy	115 157 (70.7)	6366 (100.0)	158 (527)	213 (556)
Viral URI				
Total	630 381 (100.0)	87 985 (100.0)	480 (2605)	531 (4251)
Inpatient medical	1546 (0.2)	267 (0.3)	53 (2239)	74 (3976)
Emergency department	22 367 (3.5)	2947 (3.3)	56 (463)	42 (437)
Outpatient medical	624 443 (99.1)	87 103 (99.0)	310 (946)	322 (1039)
Outpatient pharmacy	252 277 (40.0)	87 981 (100.0)	61 (589)	93 (817)
Bronchiolitis				
Total	19 222 (100.0)	1994 (100.0)	783 (2362)	704 (1506)
Inpatient medical	214 (1.1)	13 (0.6)	147 (1967)	64 (1039)
Emergency department	1367 (7.1)	136 (6.8)	114 (529)	74 (515)
Outpatient medical	19 006 (98.9)	1970 (98.8)	476 (1002)	481 (817)
Outpatient pharmacy	10 119 (52.6)	1994 (100.0)	45 (360)	85 (358)
Bronchitis				
Total	20 886 (100.0)	49 378 (100.0)	651 (2327)	515 (2406)
Inpatient medical	52 (0.2)	120 (0.2)	59 (1786)	57 (1968)
Emergency department	1022 (4.9)	2312 (4.7)	177 (837)	47 (456)
Outpatient medical	20 491 (98.1)	48 350 (97.9)	331 (1007)	299 (932)
Outpatient pharmacy	11 521 (55.2)	49 375 (100.0)	83 (374)	112 (719)
Nonsuppurative OM				
Total	61 563 (100.0)	62 160 (100.0)	643 (2231)	480 (1989)
Inpatient medical	143 (0.2)	155 (0.2)	46 (1449)	41 (1508)
Emergency department	2106 (3.4)	2227 (3.6)	55 (438)	38 (384)
Outpatient medical	61 036 (99.1)	61 528 (99.0)	495 (1436)	334 (821)
Outpatient pharmacy	20 426 (33.2)	62 158 (100.0)	47 (506)	66 (634)

Abbreviations: OM, otitis media; URI, upper respiratory infection.

The sum of attributable expenditures of inappropriate prescriptions in the MarketScan study population is presented by infection type and setting in eTable 15 in the Supplement. Table 3 and eTable 16 in the Supplement present the national annual expenditure estimates of inappropriate antibiotic treatment in the pediatric commercially insured population, which were highest for suppurative OM ($25.3 million), pharyngitis ($21.3 million), and viral URI ($19.1 million).

**Table 3.** Annual National Attributable 30-Day Expenditures of Inappropriate Antibiotic Prescriptions Among the US Commercially Insured Population, Aged 6 Months to 17 Years^a^

Index diagnoses	Attributable expenditures, 2018 US $				
	Inpatient medical	Emergency department	Outpatient medical	Outpatient pharmacy	Total
Bacterial infections (primary analysis)					
Suppurative OM	1 235 313	777 904	7 846 200	15 441 487	25 300 317
Pharyngitis	−750 188	2 564 653	6 706 388	12 752 577	21 271 338
Sinusitis	−503 873	277 773	407 416	6 899 358	7 078 513
Viral infections (secondary analysis)					
Influenza	−98 806	−53 888	1 132 300	615 754	1 594 541
Viral URI	5 430 897	439 555	8 243 074	5 023 360	19 132 099
Bronchiolitis	−334 451	48 984	88 877	159 028	−37 871
Bronchitis	1 059 296	−2 593 873	−4 624 124	2 988 452	−3 173 797
Non-suppurative OM	−16 270	−962 935	−17 980 659	3 569 023	−15 395 644

Abbreviations: OM, otitis media; URI, upper respiratory infection.

^a^ Bronchiolitis cohort was restricted to ages 6 months to 3 years; bronchitis cohort was restricted to ages 5 to 17 years. The 95% confidence intervals are presented in eTable 16 in the Supplement.

### Subgroup and Sensitivity Analyses

Results of the safety analyses for asthma and allergy and the asthma exacerbation subset were consistent with results for viral conditions, for which appropriate treatment was defined as the absence of an antibiotic prescription (eTables 17-22 and eFigures 4-6 in the Supplement). An antibiotic prescription to treat asthma and allergy was associated with increased expenditures (weighted mean total attributable expenditure, $246 [95% CI, $147-$327]); results were null and imprecise for asthma exacerbation (eTable 21 and eFigure 6 in the Supplement). We did not observe meaningful differences in calculated expenditures in sensitivity analyses that accounted for inappropriate antibiotic duration; extended follow-up from 30 to 90 days; or excluded HMO and POS with capitation plans (eTable 23 in the Supplement).

## Discussion

We conducted a national study of the safety and attributable expenditures associated with inappropriate outpatient antibiotic prescriptions for the treatment of several common bacterial and viral infections among children with commercial insurance. Inappropriate antibiotic prescriptions were associated with increased risk of ADEs, including *C. difficile* infection (suppurative OM, pharyngitis, and sinusitis cohorts), severe allergic reaction (suppurative OM cohort), and skin rash (viral URI and nonsuppurative OM cohorts). The 30-day all-cause attributable expenditures associated with inappropriate prescriptions were substantial on both the individual and national levels (eg, $56 per patient and $25.3 million nationally for suppurative OM).

The present study also broadens the evidence on pediatric antibiotic safety by quantifying the risks of individual ADEs associated with inappropriate antibiotics. Gerber and colleagues^40^ found that broad- vs narrow-spectrum antibiotics were associated with higher risk of a composite ADE outcome in children diagnosed with acute OM and similar risk in smaller cohorts of children diagnosed with sinusitis or pharyngitis. Our work builds on this study by estimating the risk of individual ADEs among children with bacterial infections as well as among children with viral infections, for whom antibiotics are inappropriate.

Our study fills a critical evidence gap by quantifying the increased expenditures associated with inappropriate antibiotic prescriptions for several common pediatric infections. Previous studies have calculated overall national antibiotic-related expenditures^41,42^ as well as antibiotic expenditures for influenza^9^ and upper respiratory infection.^10^ Our comparative expenditure analyses extend beyond the index prescription and incorporate downstream expenditures. Notably, inappropriate prescriptions were associated with higher health care expenditures for all 3 bacterial infections under study, higher or similar expenditures for 4 of 5 viral infections, and higher expenditures for noninfectious asthma and allergy.

One possible explanation for the association between inappropriate antibiotic prescriptions and larger expenditures is the higher ADE risk among recipients of inappropriate antibiotic prescriptions, which may lead to avoidable health care encounters. These encounters present additional opportunities for testing, treatment, and referrals, cascades of care that may not lead to clinically meaningful outcomes yet are associated with patient harms and monetary costs.^43,44^ Our estimates of ADE-associated expenditures represented only a small proportion of all-cause expenditures, possibly because of patients with milder ADEs choosing not to seek care, and thus, having no billable medical encounter. This phenomenon was demonstrated by a pediatric study that identified 10 times more ADEs via telephone calls to families vs manual review of electronic health record data.^40^ In the event of a cascade of care, it is possible that a minor ADE may not be recorded as a diagnosis on the claim and thus would be excluded from the ADE-attributable expenditures. Even in the absence of a billable encounter, health care providers commonly prescribe treatments for ADEs after telephone or telemedicine consultation, which may explain the higher all-cause outpatient pharmacy expenditures observed in our study.^45^

We observed widespread use of inappropriate antibiotics, consistent with previous studies^1,8^ and contrary to guidance by the US Centers for Disease Control and Prevention to reduce inappropriate antibiotic prescriptions in outpatient settings.^46,47^ Given our findings on increased patient harms and expenditures, these results warrant a call to action to key stakeholders for widespread adoption of outpatient antibiotic stewardship programs. Our study identifies suppurative OM, pharyngitis, sinusitis, and viral URIs as likely high yield targets for stewardship efforts, which could generate meaningful reductions in inappropriate antibiotic prescribing practices. Future reductions in inappropriate antibiotic prescribing will require engagement with payers, policy makers, quality improvement organizations, and patient advocacy groups. From the payer perspective, inappropriate antibiotics are a prime target for reducing health care expenditures and wasted resources,^48,49^ as antibiotics are the most commonly prescribed medication among children.^50^

### Limitations

Our findings are subject to limitations. First, owing to the nonrandomized nature of the exposure, the results may be susceptible to bias due to residual confounding. We attempted to reduce potential confounding using several established epidemiologic methods, including an active comparator new-user design,^18,19,20^ restriction of study population to otherwise healthy children,^18^ and propensity score methods.^51,52,53,54,55,56^ Furthermore, the null findings in the negative control safety analyses suggest that residual confounding was minimal.^33^ Second, cohort eligibility was based on diagnosis codes and the presence or absence of a same-day antibiotic prescription dispensing, but we cannot rule out potential misclassification of viral infections as bacterial infections, or vice versa, because of misdiagnosis by health care providers. For example, children with nonsuppurative OM, a viral condition for which antibiotics are not indicated, had lower health care expenditures if they inappropriately received an antibiotic. This finding is likely because of misidentification or incorrect coding by health care professionals of suppurative OM as nonsuppurative OM. Third, we did not account for history of antibiotic allergies or intolerances; therefore, some antibiotics deemed inappropriate may have been misclassified. Fourth, our short-term individual-level estimates of attributable health care expenditures are conservative since they do not incorporate over-the-counter treatments for ADEs or downstream medical consequences of antibiotic exposure (eg, antibiotic-resistant infections, eczema).^4,57,58^ Fifth, our national expenditure results are underestimates because they only account for children with commercial insurance (approximately 55% of the national pediatric population)^59^ and are further limited to recipients of same-day antibiotics (ie, not delayed antibiotic prescriptions). Furthermore, MarketScan is limited to commercially insured children and also overrepresents residents from the South and underrepresents residents of the West; thus, results may not be generalizable to other populations.^60^

## Conclusions

This national study underscores the negative health and financial consequences associated with inappropriate antibiotic prescriptions to treat common outpatient bacterial and viral infections in children. These findings are critical to inform decisions by health care stakeholders—including patient advocacy groups, public and private payers, and health care administrators—to implement widespread antimicrobial stewardship activities in outpatient settings to reduce antibiotic-related harms and expenditures.

## Notes

Supplement. **eMethods.** Definition of Inappropriate Antibiotic Duration for Bacterial Infections, Statistical Analysis **eTable 1. **Diagnosis Codes to Identify Eligible Patients for Pediatric Cohorts **eTable 2. **Medications to Identify Pediatric Patients for Exclusion **eTable 3. **Codes to Identify Pregnancy, Mechanical Ventilation, Hematologic or Solid Organ Malignant Neoplasms, and Hematologic or Immunologic Conditions for Exclusion **eTable 4. **Codes to Identify Pediatric Patients with Viral or Bacterial Infections for Exclusion **eTable 5. **Medications to Identify Index Oral Antibiotic Treatment **eTable 6. **Codes and Timing to Identify Adverse Drug Events for Comparative Safety Analyses **eTable 7. **Codes to Identify Baseline Characteristics **eTable 8. **Diagnosis Codes to Identify Elixhauser Comorbidities **eTable 9. **Distribution of Index Antibiotic Agents Prescribed to Children by Infection Type **eTable 10.** Additional Selected Baseline Characteristics of Children Diagnosed with Infections of Interest **eTable 11. **Number of Exclusions For Adverse Drug Event Outcomes That Occurred Within 30 Days Prior to the Index Date **eTable 12. **Unadjusted and Propensity Score–Weighted Hazard Ratio Estimates of Adverse Drug Events Following Inappropriate vs Appropriate Antibiotic Prescriptions Among Pediatric Patients **eTable 13. **Inverse Probability Of Treatment–Weighted 30-Day All Cause and Adverse Drug Event–Related Attributable Expenditure Estimates of Inappropriate Antibiotic Prescriptions Among Children by Setting **eTable 14. **Inverse Probability of Treatment–Weighted 30-Day Adverse Drug Event–Related Health Care Utilization and Total Per-Patient Expenditure Estimates of Inappropriate Antibiotic Prescriptions Among Children **eTable 15. **Total 30-Day Attributable Expenditure Estimates of Inappropriate Antibiotic Prescriptions in 2017 Pediatric MarketScan Study Population, Age 6 Months to 17 Years **eTable 16.** Confidence Intervals for Annual National Attributable 30-Day Expenditures of Inappropriate Antibiotic Prescriptions Among the US Commercially Insured Population, Age 6 Months to 17 Years **eTable 17. **Baseline Characteristics of Children Diagnosed with a Noninfectious Clinical Condition **eTable 18. **Distribution of Index Antibiotic Agents Prescribed to Children by Noninfectious Clinical Condition **eTable 19. **Number of Exclusions For Adverse Drug Event Outcomes That Occurred Within 30 Days Prior to the Index Date by Noninfectious Clinical Condition **eTable 20. **Unadjusted and Propensity Score–Weighted Hazard Ratio Estimates of Adverse Drug Events Following Inappropriate vs Appropriate Antibiotic Prescriptions Among Pediatric Patients by Noninfectious Clinical Condition **eTable 21.** Inverse Probability of Treatment–Weighted 30-Day Health Care Utilization and All-Cause and Adverse Drug Event–Related Total Per-Patient and Attributable Expenditure Estimates of Inappropriate Antibiotic Prescriptions Among Children by Noninfectious Clinical Condition **eTable 22. **Total Attributable Expenditures of Inappropriate Antibiotic Prescriptions Among Children by Noninfectious Clinical Condition **eTable 23.** Sensitivity Analyses for Inverse Probability of Treatment–Weighted All-Cause Attributable Expenditure Estimates of Inappropriate Antibiotic Prescriptions Among Children by Condition **eFigure 1. **Derivation of Pediatric Infection Cohort in MarketScan Commercial Database (Index Events April 1, 2016, to September 30, 2018) **eFigure 2. **Standardized Mean Differences of Patient- and Provider-Level Characteristics Between Treatment Groups, in the Unweighted and Weighted Pediatric Populations, for Acute Kidney Failure Outcome Cohort **eFigure 3. **Propensity Score–Weighted Hazard Ratio Estimates of Additional Adverse Drug Events Following Inappropriate vs Appropriate Antibiotic Prescriptions Among Pediatric Patients **eFigure 4. **Standardized Mean Differences of Patient- and Provider-Level Characteristics Between Treatment Groups, in the Unweighted and Weighted Populations of Children with Asthma and Allergy or Asthma Exacerbation, for Acute Kidney Failure Safety Outcome Cohort **eFigure 5. **Propensity Score–Weighted Hazard Ratio Estimates of Adverse Drug Events Following Inappropriate vs Appropriate Antibiotic Prescriptions Among Asthma or Allergy and Asthma Exacerbation Pediatric Cohorts **eFigure 6. **Weighted 30-Day Attributable Expenditures of Inappropriate Antibiotic Prescriptions for Asthma or Allergy and Asthma Exacerbation Pediatric Cohorts
eReferences.
