# Error in Figure 1

**DOI:** 10.1001/jamanetworkopen.2022.21479

**Published:** 2022-06-17

In the Original Investigation titled “Association of Inappropriate Outpatient Pediatric Antibiotic Prescriptions With Adverse Drug Events and Health Care Expenditures,”^1^ published May 26, 2022, there was an error in Figure 1. The label to the left of the vertical line indicating a weighted hazard ratio of 1 should have read “Inappropriate agent nonharmful,” and the label to the right of the line should have read “Inappropriate agent harmful.” This article has been corrected.^1^

## References

[1] Butler AM, Brown DS, Durkin MJ, . Association of inappropriate outpatient pediatric antibiotic prescriptions with adverse drug events and health care expenditures. JAMA Netw Open. 2022;5(5):e2214153. doi:10.1001/jamanetworkopen.2022.14153 35616940 PMC9136626

