# Supplementary material for: Association of Inappropriate Outpatient Pediatric Antibiotic Prescriptions With Adverse Drug Events and Health Care Expenditures
Source: JAMA Netw Open. 2022 May 26;5(5):e2214153. doi: 10.1001/jamanetworkopen.2022.14153 (PMC9136626; doi:10.1001/jamanetworkopen.2022.14153)
Supplement: Supplement. — eMethods. Definition of Inappropriate Antibiotic Duration for Bacterial Infections, Statistical Analysis eTable 1. Diagnosis Codes to Identify Eligible Patients for Pediatric Cohorts eTable 2. Medications to Identify Pediatric Patients for Exclusion eTable 3. Codes to Identify Pregnancy, Mechanical Ventilation, Hematologic or Solid Organ Malignant Neoplasms, and Hematologic or Immunologic Conditions for Exclusion eTable 4. Codes to Identify Pediatric Patients with Viral or Bacterial Infections for Exclusion eTable 5. Medications to Identify Index Oral Antibiotic Treatment eTable 6. Codes and Timing to Identify Adverse Drug Events for Comparative Safety Analyses eTable 7. Codes to Identify Baseline Characteristics eTable 8. Diagnosis Codes to Identify Elixhauser Comorbidities eTable 9. Distribution of Index Antibiotic Agents Prescribed to Children by Infection Type eTable 10. Additional Selected Baseline Characteristics of Children Diagnosed with Infections of Interest eTable 11. Number of Exclusions For Adverse Drug Event Outcomes That Occurred Within 30 Days Prior to the Index Date eTable 12. Unadjusted and Propensity Score–Weighted Hazard Ratio Estimates of Adverse Drug Events Following Inappropriate vs Appropriate Antibiotic Prescriptions Among Pediatric Patients eTable 13. Inverse Probability Of Treatment–Weighted 30-Day All Cause and Adverse Drug Event–Related Attributable Expenditure Estimates of Inappropriate Antibiotic Prescriptions Among Children by Setting eTable 14. Inverse Probability of Treatment–Weighted 30-Day Adverse Drug Event–Related Health Care Utilization and Total Per-Patient Expenditure Estimates of Inappropriate Antibiotic Prescriptions Among Children eTable 15. Total 30-Day Attributable Expenditure Estimates of Inappropriate Antibiotic Prescriptions in 2017 Pediatric MarketScan Study Population, Age 6 Months to 17 Years eTable 16. Confidence Intervals for Annual National Attributable 30-Day Expenditures of Inappropriate Antibiotic Prescriptio [file jamanetwopen-e2214153-s001.pdf]

Supplemental Online Content

Butler AM, Brown DS, Durkin MJ, et al. Association of inappropriate outpatient pediatric antibiotic prescriptions with adverse drug events and health care expenditures. *JAMA Netw Open.* 2022;5(5):e2214153. doi:10.1001/jamanetworkopen.2022.14153

eMethods. Definition of Inappropriate Antibiotic Duration for Bacterial Infections, Statistical Analysis

eTable 1. Diagnosis Codes to Identify Eligible Patients for Pediatric Cohorts

eTable 2. Medications to Identify Pediatric Patients for Exclusion

eTable 3. Codes to Identify Pregnancy, Mechanical Ventilation, Hematologic or Solid Organ Malignant Neoplasms, and Hematologic or Immunologic Conditions for Exclusion

eTable 4. Codes to Identify Pediatric Patients with Viral or Bacterial Infections for Exclusion

eTable 5. Medications to Identify Index Oral Antibiotic Treatment

eTable 6. Codes and Timing to Identify Adverse Drug Events for Comparative Safety Analyses

eTable 7. Codes to Identify Baseline Characteristics

eTable 8. Diagnosis Codes to Identify Elixhauser Comorbidities

eTable 9. Distribution of Index Antibiotic Agents Prescribed to Children by Infection Type

eTable 10. Additional Selected Baseline Characteristics of Children Diagnosed with Infections of Interest

eTable 11. Number of Exclusions For Adverse Drug Event Outcomes That Occurred Within 30 Days Prior to the Index Date

eTable 12. Unadjusted and Propensity Score–Weighted Hazard Ratio Estimates of Adverse Drug Events Following Inappropriate vs Appropriate Antibiotic Prescriptions Among Pediatric Patients

eTable 13. Inverse Probability Of Treatment–Weighted 30-Day All Cause and Adverse Drug Event–Related Attributable Expenditure Estimates of Inappropriate Antibiotic Prescriptions Among Children by Setting

eTable 14. Inverse Probability of Treatment–Weighted 30-Day Adverse Drug Event–Related Health Care Utilization and Total Per-Patient Expenditure Estimates of Inappropriate Antibiotic Prescriptions Among Children

eTable 15. Total 30-Day Attributable Expenditure Estimates of Inappropriate Antibiotic Prescriptions in 2017 Pediatric MarketScan Study Population, Age 6 Months to 17 Years

eTable 16. Confidence Intervals for Annual National Attributable 30-Day Expenditures of Inappropriate Antibiotic Prescriptions Among the US Commercially Insured Population, Age 6 Months to 17 Years

© 2022 Butler AM et al. *JAMA Network Open.*

eTable 17. Baseline Characteristics of Children Diagnosed with a Noninfectious Clinical Condition

eTable 18. Distribution of Index Antibiotic Agents Prescribed to Children by Noninfectious Clinical Condition

eTable 19. Number of Exclusions For Adverse Drug Event Outcomes That Occurred Within 30 Days Prior to the Index Date by Noninfectious Clinical Condition

eTable 20. Unadjusted and Propensity Score–Weighted Hazard Ratio Estimates of Adverse Drug Events Following Inappropriate vs Appropriate Antibiotic Prescriptions Among Pediatric Patients by Noninfectious Clinical Condition

eTable 21. Inverse Probability of Treatment–Weighted 30-Day Health Care Utilization and All-Cause and Adverse Drug Event–Related Total Per-Patient and Attributable Expenditure Estimates of Inappropriate Antibiotic Prescriptions Among Children by Noninfectious Clinical Condition

eTable 22. Total Attributable Expenditures of Inappropriate Antibiotic Prescriptions Among Children by Noninfectious Clinical Condition

eTable 23. Sensitivity Analyses for Inverse Probability of Treatment–Weighted All-Cause Attributable Expenditure Estimates of Inappropriate Antibiotic Prescriptions Among Children by Condition

eFigure 1. Derivation of Pediatric Infection Cohort in MarketScan Commercial Database (Index Events April 1, 2016, to September 30, 2018)

eFigure 2. Standardized Mean Differences of Patient- and Provider-Level Characteristics Between Treatment Groups, in the Unweighted and Weighted Pediatric Populations, for Acute Kidney Failure Outcome Cohort

eFigure 3. Propensity Score–Weighted Hazard Ratio Estimates of Additional Adverse Drug Events Following Inappropriate vs Appropriate Antibiotic Prescriptions Among Pediatric Patients

eFigure 4. Standardized Mean Differences of Patient- and Provider-Level Characteristics Between Treatment Groups, in the Unweighted and Weighted Populations of Children with Asthma and Allergy or Asthma Exacerbation, for Acute Kidney Failure Safety Outcome Cohort

eFigure 5. Propensity Score–Weighted Hazard Ratio Estimates of Adverse Drug Events Following Inappropriate vs Appropriate Antibiotic Prescriptions Among Asthma or Allergy and Asthma Exacerbation Pediatric Cohorts

eFigure 6. Weighted 30-Day Attributable Expenditures of Inappropriate Antibiotic Prescriptions for Asthma or Allergy and Asthma Exacerbation Pediatric Cohorts

eReferences.

This supplemental material has been provided by the authors to give readers additional information about their work.

© 2022 Butler AM et al. *JAMA Network Open.*

eMethods. Definition of Inappropriate Antibiotic Duration for Bacterial Infections, Statistical Analysis

Subgroup and Sensitivity Analysis: Definition of Inappropriate Antibiotic Duration for Bacterial Infections

Inappropriate antibiotic duration was based on guideline recommendations. For suppurative OM, appropriate duration was amoxicillin for 10 days for children <2 years, 7 days for children 2–5 years, and 5–7 days for children ≥6 years.[1] For pharyngitis, appropriate duration was amoxicillin or penicillin for 10 days.[2] For sinusitis, appropriate duration was amoxicillin or amoxicillin-clavulanate for 10–14 days.[3] Note that unusual durations of <5 days or >14 days were excluded for this study; therefore inappropriate duration was 5–14 days and outside the recommended duration per infection.

Statistical Analysis

We used stabilized inverse probability of treatment (IPT) weights to balance treatment groups within each cohort with respect to potential confounding factors. We used logistic regression to estimate the propensity of appropriate (versus inappropriate) antibiotic agent, conditional on baseline covariates. Age and average monthly expenditures were modeled using restricted cubic splines. Propensity scores were used to create weighted cohorts to estimate the treatment effects in the total population (the average treatment effect). To minimize the impact of large weights due to children being treated contrary to prediction, we applied asymmetric trimming of the propensity score as needed to account for areas of non-overlap or by percentiles (i.e., 0.5th percentile of the appropriately treated and 99.5th percentile of the inappropriately treated trimmed in the whole cohort); and propensity scores were re-estimated after these exclusions.[4,5] After weighting, we assessed the balance of observed covariates between treatment groups; absolute standardized mean differences < 10% in the weighted population were considered adequate.[6]

© 2022 Butler AM et al. *JAMA Network Open.*

eTable 1. Diagnosis Codes to Identify Eligible Patients for Pediatric Cohorts[a]

	ICD-10-CM
**Primary analysis: Bacterial infections**	
Suppurative OM	H66.001-H66.019, H66.3X1-H67.9
Pharyngitis	A38.0-A38.9, J02.0-J03.91
Sinusitis	J01.00-J01.91, J32.0-J32.9
**Secondary analysis: Viral infections**	
Bronchiolitis	J21.0-J21.9
Bronchitis	J20.0-J20.9, J40
Influenza	J09.X1-J11.89
Viral URI	J00, J04.0-J06.9, R05
Non-suppurative OM	H65.00-H65.93, H68.001-H68.029
**Subgroup analysis: Noninfectious clinical conditions**	
Asthma/allergy	J30.0-J30.9, J44.0-J45.998, T78.40XA, T78.49XA
Asthma exacerbation	J44.1, J45.21, J45.31, J45.41, J45.51, J45.901

Abbreviations: ICD-10-CM, International Classification of Diseases, Tenth Revision, Clinical Modification; OM, otitis media; URI, upper respiratory infection.

[a] Eligible diagnoses required a claimline payment > $0.

© 2022 Butler AM et al. *JAMA Network Open.*

eTable 2. Medications to Identify Pediatric Patients for Exclusion

Conditions	Medication Names	HCPCS Codes
Anti-infectives[a]	amoxicillin, amoxicillin/clavulanate, amikacin, ampicillin, ampicillin/sulbactam, azithromycin, aztreonam, cefaclor, cefazolin, cefadroxil, cefepime, cefdinir, cefditoren, cefixime, cefotetan, cefoxitin, cefpodoxime, cefprozil, ceftibuten, ceftazidime, ceftazidime/avibactam, ceftriaxone, cefuroxime, cephalexin, ceftaroline, ciprofloxacin, clarithromycin, clindamycin, dalbavancin, daptomycin, dicloxacillin, doripenem, doxycycline, ertapenem, erythromycin, ethambutol, fosfomycin, gatifloxacin, gemifloxacin, gentamicin, imipenem/cilastain, levofloxacin, linezolid, methicillin, metronidazole, minocycline, moxifloxacin, nafcillin, nitrofurantoin, norfloxacin, ofloxacin, oxacillin, penicillin, piperacillin/tazobactam, quinpristin/dalfopristin, rifabutin, rifampin, tedizolid, telithromycin, tetracycline, ticarcillin/clavulanic acid, tigecycline, tobramycin, trimethoprim, trimethoprim/sulfamethoxazole, vancomycin	C9479, J0290, J0295, J0456, J0558, J0561, J0694, J0696, J0697, J0744, J0878, J1364, J1580, J1590, J1956, J2020, J2265, J2280, J2510, J2540, J3243, J3260, J3370, J7342, J7682, J7685, Q0144, S0030, S0034, S0039, S0077
Antibiotic ear drops (exclude for non-suppurative otitis media only)	bacitracin zinc/neomycin/polymyxin b sulfate, bacitracin zinc/polymyxin b sulfate, ciprofloxacin hydrochloride, ciprofloxacin hydrochloride/dexamethasone, ciprofloxacin hydrochloride/fluocinolone acetonide, ciprofloxacin hydrochloride/hydrocortisone, clindamycin phosphate, colistin sulf/hc ace/neomycin sulf/thonzonium brom, dexamethasone/neomycin sulfate/polymyxin b sulfate, erythromycin, gentamicin sulfate, hc/neomycin sulf/polymyxin b sulf, moxifloxacin hydrochloride, nystatin, ofloxacin, polymyxin b sulfate/trimethoprim sulfate, tobramycin	
Autoimmune conditions, steroids, or other immunosuppressants	abatacept, adalimumab, alefacept, azathioprine, balsalazide disodium, belimumab, canakinumab, certolizumab pegol, cevimeline, cyclophosphamide, cyclosporine, dexamethasone[a], dexamethasone acetate[a], dexamethasone sodium phosphate[a], dexamethasone micronized[a], efalizumab, etanercept, golimumab, immune globulin, infliximab, leflunomide, mercaptopurine, mesalamine, methotrexate, methylprednisolone[a], methylprednisolone acetate[a], methylprednisolone sodium succinate[a], methylprednisolone micronized[a], mycophenolate mofetil, mycophenolate sodium, omalizumab, prednisolone[a], prednisolone acetate[a], prednisolone sodium phosphate[a], prednisone[a], rituximab, sirolimus, sulfasalazine, tacrolimus, tocilizumab, tofacitinib, ustekinumab	C9487, J0129, J0135, J0215, J0490, J0638, J0717, J0850, J1020[a], J1030[a], J1040[a], J1094[a], J1100[a], J1438, J1459, J1555, J1556, J1557, J1559, J1561, J1562, J1566, J1568, J1569, J1572, J1575, J1599, J1602, J1720, J1745, J2323, J2357, J2650[a], J2920[a], J2930[a], J3262, J3357, J3358, J3380, J7500, J7501, J7502, J7503, J7504,

© 2022 Butler AM et al. *JAMA Network Open.*

Conditions	Medication Names	HCPCS Codes
		J7506[a], J7507, J7508, J7509[a], J7510[a], J7511, J7512[a], J7515, J7517, J7520, J7525, J8530, J8540[a], J8610, J9070, J9250, J9260, J9310, J9330, Q5102, Q9989, S0108
HIV/AIDS	abacavir, abacavir/lamivudine, abacavir/lamivudine/zidovudine, abacavir/dolutegravir/lamivudine, atazanavir, atazanavir/cobicistat, cobicistat, cobicistat/elvitegravir/emtricitabine/tenofovir, dapsone, darunavir, darunavir/cobicistat, dolutegravir, efavirenz, efavirenz/emtricitabine/tenofovir, elvitegravir/cobicistat/emtricitabine/tenovofir disporoxul fumarate, emtricitabine/tenofovir alafenamide, emtricitabine and tenofovir disoproxil fumarate, emtricitabine/rilpivirine/tenofovir, enfuvirtide, etravirine, fosamprenavir, indinavir, lamivudine, lamivudine/zidovudine, lamivudine and tenofovir disoproxil fumarate, lopinavir/ritonavir, maraviroc, nelfinavir, nevirapine, raltegravir, rilpivirine, ritonavir, saquinavir, tipranavir, tenofovir, zidovudine	
Malignancy or myelodysplastic syndromes	aflibercept, aldesleukin, arsenic trioxide, asparaginase, atezolizumab, avelumab, azacitidine, belinostat, bendamustine, bevacizumab, bleomycin, blinatumomab, bortezomib, brentuximab, cabazitaxel, calaspargase, carboplatin, carfilzomib, carmustine, cemiplimab, cetuximab, cisplatin, cladribine, clofarabine, copanlisib, cyclophosphamide, cytarabine, dacarbazine, dactinomycin, daratumumab, daunorubicin, degarelix, denileukin, docetaxel, doxorubicin, durvalumab, elliott, elotuzumab, emapalumab, enfortumab, epirubicin, eribulin, etoposide, floxuridine, fludarabine, fluorouracil, fulvestrant, gemcitabine, gemtuzumab ozogamicin, goserelin, histrelin, hyaluronidase, idarubicin, ifosfamide, inotuzumab ozogamicin, ipilimumab, irinotecan, ixabepilone, leuprolide, mechlorethamine, melphalan, mesna, mitomycin, mitoxantrone, mogamulizumab, moxetumomab, necitumumab, nelarabine, nivolumab, obinutuzumab, ofatumumab, olaratumab, omacetaxine mepesuccinate, oxaliplatin, paclitaxel, panitumumab, pegaspargase, pembrolizumab, pemetrexed, pentostatin, pertuzumab, polatuzumab, porfimer, pralatrexate, ramucirumab, rituximab, romidepsin, streptozocin, tagraxofusp, temozolomide, temsirolimus, thiotepa, topotecan, trabectedin, trastuzumab, valrubicin, vinblastine, vincristine, vinorelbine	

Abbreviations: HCPCS, Healthcare Common Procedure Coding System; HIV/AIDS, human immunodeficiency virus/acquired immunodeficiency syndrome.

[a] All exclusions for 180 days of baseline, with the exception of 90 days of baseline for the anti-infectives and 30 days of baseline for dexamethasone, prednisone, and prednisolone.

© 2022 Butler AM et al. *JAMA Network Open.*

eTable 3. Codes to Identify Pregnancy, Mechanical Ventilation, Hematologic or Solid Organ Malignant Neoplasms, and Hematologic or Immunologic Conditions for Exclusion

Condition	ICD-10-CM Diagnosis Codes	ICD-10-PCS Procedure Codes	CPT/HCPCS Codes
Pregnancy	O00.0-O9A.53, Z33.1-Z37.9		
Mechanical ventilation	J95.850-J95.859, Z99.11-Z99.12	5A09358-5A0935Z, 5A09458-5A0945Z, 5A09558-5A0955Z, 5A1935Z-5A1955Z	94002-94004
Hematologic / solid organ malignancies	C00.0-C43.9, C45.0-C45.7, C46.0-C7A.098, C7B.00-C80.1, C81.00-C90.32, C96.0-C96.4, C96.9-C96.Z, D03.0-D03.9, D47.Z9, E31.21-E31.23, R18.0		J9000, J9015, J9017, J9019, J9020, J9022, J9023, J9025, J9027, J9032, J9033, J9034, J9035, J9036, J9039, J9040, J9041, J9042, J9043, J9044, J9045, J9047, J9050, J9055, J9057, J9060, J9065, J9070, J9098, J9100, J9118, J9119, J9120, J9130, J9145, J9150, J9151, J9153, J9155, J9160, J9171, J9173, J9175, J9176, J9177, J9178, J9179, J9181, J9185, J9190, J9198, J9200, J9201, J9202, J9203, J9204, J9205, J9206, J9207, J9208, J9209, J9210, J9211, J9217, J9218, J9219, J9225, J9226, J9228, J9229, J9230, J9245, J9246, J9261, J9262, J9263, J9264, J9266, J9267, J9268, J9269, J9271, J9280, J9285, J9293, J9295, J9299, J9301, J9302, J9303, J9305, J9306, J9307, J9308, J9309, J9311, J9312, J9313, J9315, J9320, J9328, J9330, J9340, J9351, J9352, J9354, J9355, J9356, J9357, J9358, J9360, J9370, J9371, J9390, J9395, J9400, J9600
**Hematologic/immunologic conditions**			
Hereditary anemias	D55-D58		
Aplastic anemias	D60.0-D61.9, D71		
Hereditary immunodeficiency	D80-D89, D72.0, M30.3, M35.9		
Coagulation/hemorrhagic	D66, D68.2, D69.41-D69.49		
Leukopenia	D70.0, D70.4		

© 2022 Butler AM et al. *JAMA Network Open.*

Condition	ICD-10-CM Diagnosis Codes	ICD-10-PCS Procedure Codes	CPT/HCPCS Codes
Hemophagocytic Syndromes	D76.1-D78.89		
Sarcoidosis	D86.9		
AIDS	B20		
Polyarteritis nodosa and related conditions	M30.0, M31.0-M31.1, M31.30, M31.4, M31.6l		
Diffuse diseases of connective tissue	M32.10, M33.90, M34.0-M34.1, M34.9		
Spleen resection		07TP0ZZ-07TP4ZZ	
Transplantation	Z48.21-Z48.298, Z94.0-Z94.9		
Rheumatologic conditions	M04.2-M06.9, M08.00-M08.99, M32.0-M33.19, M33.90-M33.99, M35.00-M35.1, M35.5, M35.8-M36.0, M36.8, M45.0-M45.9, M48.8X1-M48.8X9		
Inflammatory bowel disease	K40.10-K40.11, K40.40-K40.41, K50.10-K51.919, K59.31		

Abbreviations: AIDS, acquired immunodeficiency syndrome; CPT, Current Procedural Terminology; HCPCS, Healthcare Common Procedure Coding System; ICD-10-CM, International Classification of Diseases, Tenth Revision, Clinical Modification; ICD-10-PCS, International Classification of Diseases, Tenth Revision, Procedure Coding System.

© 2022 Butler AM et al. *JAMA Network Open.*

eTable 4. Codes to Identify Pediatric Patients with Viral or Bacterial Infections for Exclusion

Infection	ICD-10-CM Diagnosis Codes
Septicemia	A02.1, A20.7, A22.7, A39.2-A39.4, A39.51, A40.0-A41.9, A42.7, A48.3, I26.01, I26.90, I33.0-I33.9, I38-I40.0, I76, M32.11, R78.81, T80.211A
Clinical sepsis	A26.7, A32.7, B37.7, R65.20-R65.21, T81.12XA
Pneumonia	A02.22, A20.2, A21.2, A22.1, A37.01, A37.11, A37.81, A37.91, A42.0, A43.0, A48.1, A70, A78, B25.0, B37.1, B38.0-B38.2, B39.0-B39.2, B40.0-B40.2, B41.0, B42.0, B44.0-B44.1, B45.0, B46.0, B77.81, J09.X1, J10.00-J10.08, J11.00-J11.08, J13-J18.1, J18.8-J18.9, J85.0-J85.2, J86.0-J86.9, J95.851
Skin and soft tissue infection (SSTI)	A20.1, A21.0, A22.0, A28.1, A36.3, A42.2, A42.89-A42.9, A43.1-A48.0, A48.52, A68.0-A68.9, A69.20-A69.29, A77.0-A77.8, A79.0-A79.9, B47.0-B47.9, B60.0, B60.8-B64, B78.1, E83.2, K68.12, L01.00-L03.212, L03.221-L05.02, L08.0-L08.9, L73.2, L88, L92.8, L98.0, L98.3, M60.000-M60.09, M65.00-M65.08, M67.20-M67.29, M67.80-M67.89, M71.00-M71.09, M71.80-M71.89, M72.6, N98.0, O91.011-O91.23, T79.8XXA, T80.212A, T80.218A, T80.219A, T80.22XA, T80.29XA, T87.40-T87.44, T88.0XXA
Surgical site infection (SSI)	K95.01, K95.81, T81.4XXA-T81.4XXS, T82.6XXA-T82.7XXS, T83.61XA-T83.6XXS, T84.50XA-T84.7XXS, T85.71XA-T85.79XS, T86.842
Bone infection	A02.23-A02.24, A18.01, A18.03, A25.1, A39.83-A39.84, A54.40-A54.49, H05.021-H05.029, J85.3, M00.00-M01.X9, M46.20-M46.28, M86.00-M86.9, M90.80-M90.89
Organ infection	A02.21, A18.84, A27.81, A36.81, A39.0-A39.1, A39.50, A39.52-A39.81, A39.89, A42.81-A42.82, A51.41, D73.0, D73.3-D73.5, D73.89, E06.0, E23.6, E24.1, E32.1, G00.0-G01, G03.9, G04.2, G05.3-G07, G09, I30.1-I30.9, I32, M32.12
Female pelvic infection	A34, A51.0-A51.39, A51.42-A51.9, A54.00-A54.39, A54.6-A58, A63.8-A64, M02.30-M02.39, N34.1-N34.3, N70.01-N73.5, N73.9, N75.1, N76.0-N76.4, O03.0, O03.37, O03.5, O03.87, O04.5, O04.87, O07.0, O07.37, O08.0, O08.82, O23.511-O23.93, O41.1010-O41.1499, O75.3, O85, O86.11-O86.29, O86.81-O86.89, O98.111-O98.33, O98.611-O98.63, O98.811-O98.83, O99.830-O99.835
Gastroenteritis	A00.0-A02.0, A02.20, A02.25-A04.6, A04.71-A05.9, A09, A21.3, A22.2
Intraabdominal abscess / peritonitis	A42.1, B25.2, K20.8, K35.2-K35.891, K50.014, K50.114, K50.814, K50.914, K51.014, K51.214, K51.314, K51.414, K51.514, K51.814, K51.914, K57.00-K57.93, K61.0-K61.4, K63.0, K65.0-K65.2, K65.8-K65.9, K67-K68.11, K68.19-K68.9, K75.0, K80.00-K80.01, K80.12-K80.13, K80.42-K80.43, K80.46-K80.47, K80.62-K80.63, K80.66-K80.67, K81.0, K81.2, K85.0-K85.92, K94.02, K94.12, K94.22, K94.32
Tonsillitis	A36.0-A36.2, A36.82-A36.84, A36.86-A37.00, A37.10, A37.80, A37.90, A38.0-A38.9, A54.5, A71.0-A74.0, H05.011-H05.019, H05.031-H05.039, H16.311-H16.319, H70.201-H70.229, J34.0-J34.1, J34.89-J35.03, J36, J38.2-J38.3, J38.7-J39.2, J39.8-J39.9, J95.02
Otitis	H60.00-H60.329, H60.391-H60.399, H61.90-H62.8X9, H70.001-H70.13, H73.001-H73.23
Oral infections	K04.0-K04.02, K04.5-K04.7, K05.00-K05.329, K11.3, K12.2, K13.0, K14.0, M27.2
Urinary tract infection (UTI)	A18.14, A36.85, N10-N12, N13.6, N15.1, N15.9-N16, N28.84-N28.86, N30.00-N30.01, N30.20-N30.21, N30.80-N30.91, N34.0, N35.111-N35.114, N35.119-N35.12, N37-N39.0, N41.0-N41.3, N41.9, N43.1, N45.4, N48.21-N48.29, N49.0-N49.9, N51, O23.00-O23.43, O86.11, O86.13-O86.29, T83.510A, T83.511A, T83.512A, T83.518A, T83.51XA, T83.59XA

© 2022 Butler AM et al. *JAMA Network Open.*

Infection	ICD-10-CM Diagnosis Codes
Miscellaneous bacterial infections	A07.8, A15.0-A32.9, A35-A37.91, A39.0-A44.9, A48.0, A48.2-A59.9, A63.0-A79.9, A82.0-A82.9, A88.1, A96.0-A96.9, A98.3-A98.5, B07.0-B07.9, B08.1, B08.4-B08.5, B08.8, B15.0-B19.9, B25.0-B33.0, B33.20-B34.8, B45.1, B47.1-B47.9, B50.0-B64, B83.4, B85.0-B99.9, D86.0-D86.9, G00.0-G03.1, G03.8-G04.02, G04.2-G05.4, G14, G37.4, G92, H70.001-H70.93, H75.00-H75.83, H95.00-H95.199, J17, J20.0-J20.7, J36, K90.81, L08.1, L94.6, M00.00-M00.89, M02.30-M02.39, M35.2, M60.009, N34.1, R11.11
Otitis externa	B37.84, H60.00-H60.93, H62.40-H62.43

Abbreviations: ICD-10-CM, International Classification of Diseases, Tenth Revision, Clinical Modification.

© 2022 Butler AM et al. *JAMA Network Open.*

eTable 5. Medications to Identify Index Oral Antibiotic Treatment[a]

Medication Names[b,c]	HCPCS Codes
amoxicillin, amoxicillin-clavanulate, azithromycin, cefaclor, cefadroxil, cefdinir, cefditoren, cefixime, cefpodoxime, cefprozil, ceftibuten, cefuroxime, cephalexin, ciprofloxacin, clarithromycin, clindamycin, dicloxacillin, doxycycline, erythromycin, fosfomycin, gemifloxacin, levofloxacin, linezolid, metronidazole, minocycline, moxifloxacin, nitrofurantoin, norfloxacin, ofloxacin, penicillin V, rifampin, sulfamethoxazole-trimethoprim, telithromycin, tetracycline, trimethoprim, vancomycin	G9313, G9314, G9315, Q0144

Abbreviations: HCPCS, Healthcare Common Procedure Coding System.

[a] Eligible prescriptions required a payment > $0, and days' supply and metric quantity > 0.

[b] We defined 36 index oral antibiotics of interest based on the 2016 "antibiotic utilization" measure in the Healthcare Effectiveness Data and Information Set (HEDIS), which is a collection of quality measures reported by more than 90% of U.S. health insurance plans. We did not consider three antibiotics (i.e., sulfadiazine, lincomycin, ampicillin) because they are used to treat conditions not relevant to this study.

[c] For bacterial infections, antibiotics were considered appropriate if they were first-line agents per treatment guidelines and inappropriate otherwise. For viral infections and noninfectious clinical conditions, appropriate treatment was defined as no prescription for any of the 36 listed antibiotics, and a prescription of any of the 36 listed antibiotic agents was considered inappropriate. See "Antibiotic Exposure" section of the manuscript for additional details.

© 2022 Butler AM et al. *JAMA Network Open.*

eTable 6. Codes and Timing to Identify Adverse Drug Events for Comparative Safety Analyses

Adverse Drug Event	Follow-up Duration Relative to Index Date	ICD-10-CM Diagnosis Codes[a]
**Dermatologic**		
Skin rash	1-14 days	R21
Stevens-Johnson syndrome	1-14 days	L511
Toxic epidermal necrolysis	1-14 days	L51.2-L51.3
Urticaria	1-14 days	L50.0-L50.1, L50.6-L50.9
**Gastrointestinal**		
Nausea/vomiting	1-14 days	R11.0-R11.12, R11.2
Abdominal pain	1-14 days	R10.0-R10.33, R10.84-R10.9
Non-*C. difficile* diarrhea	1-30 days	K52.29-K52.3, K52.831-K52.9, R19.7
C. difficile infection	1-90 days	A04.7-A04.72
**Hypersensitivity**		
Anaphylaxis	0-2 days	T78.2XXA, T88.6XXA
Angioedema	0-2 days	H02.841-H02.849, T78.3XXA
Laryngeal edema	0-2 days	J38.4
Unspecified allergy	1-14 days	T78.40XA, T78.49XA
**Neuromuscular and skeletal**		
Tendinopathy (including tendon rupture)	1-90 days	M66.20, M66.221-M66.80, M66.821-M66.9, M75.120-M75.122, M76.50-M76.62, M76.811-M76.829, M77.9
**Kidney**		
Kidney failure	1-14 days	N17.0-N17.9

Abbreviations: ICD-10-CM, International Classification of Diseases, Tenth Revision, Clinical Modification.

[a] Diagnostic/rule out claims were not included in identifying adverse drug events.

© 2022 Butler AM et al. *JAMA Network Open.*

eTable 7. Codes to Identify Baseline Characteristics

	CPT/HCPCS
**Office visits**	
Office visits	99201-99205, 99211-99215, 99241-99245, 99381-99386, 99391-99396, 99401-99409
**Durable medical equipment**	
Oxygen equipment	E0430-E0435, E0439-E0444, E1390-E1392
Home hospital bed	E0290-E0297, E0301-E0304, E0250, E0251, E0255, E0256, E0260, E0261, E0265, E0266, E0270, E0316
Mobility aids	E0100 E0105 E0130 E0135 E0140 E0141 E0143 E0144 E0147 E0148 E0149, E0163-E0175, E0240-E0248, E0950-E1228, E1230, E1240-E1298, K0001-K0009
**Other**	
Non-SNF-related rehabilitation services	92507, 92508, 92521, 92522, 92523, 92524, 92526, 92607, 92608, 92609, 92610, 92611, 92612, 92613, 92616, 92617, 97001, 97002, 97003, 97004, 97110-97150, 97161, 97162, 97163, 97164, 97165, 97166, 97167, 97168, 97530, 97532-97546, G0129, G0151, G0152, G0153, G0157, G0158, G0159, G0160, G0161, G8699, S9128, S9129, S9131
Ambulance / life support	A0426 A0427 A0428 A0429 A0999

Abbreviations: CPT, Current Procedural Terminology; HCPCS, Healthcare Common Procedure Coding System; SNF, skilled nursing facility.

© 2022 Butler AM et al. *JAMA Network Open.*

eTable 8. Diagnosis Codes to Identify Elixhauser Comorbidities

Condition	ICD-10-CM Diagnosis Codes
Alcohol abuse	F10.10-F10.29, F10.921, F10.94-F10.99
Deficiency anemias	D50.1-D53.9, D63.0-D63.8, D64.9
Rheumatoid arthritis/ collagen vascular disease	L90.0, L94.0-L94.1, L94.3, M05.00-M06.9, M08.00-M08.99, M12.00-M12.09, M32.0-M35.1, M35.3, M35.5, M35.8-M36.0, M36.8, M45.0-M46.1, M46.50-M46.99, M48.8X1-M48.8X9, M49.80-M49.89
Chronic blood loss anemia	D50.0, O90.81, O99.011-O99.03
Chronic pulmonary disease	J40-J64, J66.0-J67.9, J68.4
Congestive heart failure	I09.81, I50.1-I50.9
Kidney failure	I12.0, I13.11-I13.2, N18.3-N19, Z49.01-Z49.32, Z91.15, Z94.0, Z99.2
Coagulopathy	D65-D68.4, D68.8-D68.9, D69.1, D69.3-D69.6, D75.82, O99.111-O99.13
Depression	F32.0-F32.3, F32.8-F33.3, F33.8-F33.9, F34.1, F43.21
Diabetes	E08.00-E13.9, O24.011-O24.33, O24.811-O24.93, P70.2
Drug abuse	F11.10-F11.29, F12.10-F12.229, F12.250-F12.29, F13.10-F13.29, F14.10-F14.29, F15.10-F15.29, F16.10-F16.29, F18.10-F18.29, F19.10-F19.29, F55.0-F55.8, O99.320-O99.325
Hypertension	I10-I16.9, I67.4, O10.011-O11.9, O16.1-O16.9
Hypothyroidism	E00.0-E00.9, E01.8-E03.3, E03.8-E03.9, E89.0
Liver disease	B18.0-B18.2, I85.00-I85.11, K70.0, K70.2-K70.9, K72.10-K74.69, K75.4-K75.81, K76.0, K76.6, K76.89-K76.9, Z94.4
Lymphoma	C81.00-C90.32, C96.0-C96.4, C96.9-C96.Z, D47.Z9
Fluid and electrolyte disorders	E86.0-E87.8
Metastatic cancer	C77.0-C79.9, C7B.00-C80.1, R18.0
Other neurological disorders	E75.00-E75.19, E75.23, E75.25, E75.29, E75.4, F84.2, G10-G12.9, G13.2-G13.8, G20, G21.4, G24.01-G24.09, G24.2, G24.8, G25.4-G25.5, G25.81, G30.0-G31.9, G32.81, G35, G36.1-G40.B19, G47.411-G47.429, G80.3, G89.0, G91.0-G91.9, G93.7, G93.89-G94, O99.350-O99.355, P91.60-P91.63, R41.0, R41.82, R47.01, R56.00-R56.9
Obesity	E66.01-E66.2, E66.8-E66.9, O99.210-O99.215, R93.9, Z68.30-Z68.45, Z68.54
Paralysis	G04.1, G80.0-G80.2, G80.4-G83.9, I69.031-I69.069, I69.131-I69.169, I69.231-I69.269, I69.331-I69.369, I69.831-I69.869, I69.931-I69.969, R53.2
Peripheral vascular disorders	I70.0-I72.9, I73.1-I73.9, I74.2-I74.4, I76, I77.1, I77.70-I77.79, I79.0-I79.8, K55.1, K55.8-K55.9, Z95.820-Z95.828

© 2022 Butler AM et al. *JAMA Network Open.*

Condition	ICD-10-CM Diagnosis Codes
Pulmonary circulation disorders	I26.01-I26.92, I26.99-I27.1, I27.81-I27.9, I28.9, T80.0XXA, T82.817A, T82.818A
Psychoses	F20.0-F20.9, F22-F31.9, F32.4-F32.5, F33.40-F33.42, F34.8-F39, F44.89, F84.3
Solid tumor without metastasis	C00.0-C43.9, C45.0-C45.7, C46.0-C76.8, C7A.00-C7A.098, D03.0-D03.9, E31.21-E31.23
Valvular disease	A52.03, I05.0-I08.9, I09.1, I09.89, I34.0-I39, Q23.0-Q23.3, Z95.2-Z95.4
Weight loss	E40-E46, E64.0, R63.4, R63.6

Abbreviations: ICD-10-CM, International Classification of Diseases, Tenth Revision, Clinical Modification.

© 2022 Butler AM et al. *JAMA Network Open.*

eTable 9. Distribution of Index Antibiotic Agents Prescribed to Children by Infection Type[a]

Antibiotic agent	Bacterial Infections			Viral Infections				
	Suppurative OM n = 601,711 (%)	Pharyngitis n = 617,215 (%)	Sinusitis n = 382,093 (%)	Influenza n = 180,996 (%)	Viral URI n = 772,040 (%)	Bronchiolitis n = 23,931 (%)	Bronchitis n = 72,407 (%)	Non-suppurative OM n = 153,852 (%)
None	0.0	0.0	0.0	96.2	88.0	91.1	29.8	52.3
Amoxicillin-clavulanate	9.9	5.8	21.5	0.2	0.8	0.6	3.1	4.2
Amoxicillin	69.0	63.3	42.6	1.3	4.2	4.5	8.1	31.9
Azithromycin	6.4	13.1	16.7	1.8	5.6	2.9	53.4	4.3
Cefdinir	12.6	7.7	14.3	0.3	0.9	0.7	2.9	5.9
Cefprozil	0.9	0.8	1.2	0.0	0.1	0.1	0.3	0.4
Cephalexin	0.4	4.8	0.8	0.1	0.2	0.1	0.4	0.3
Penicillin V	0.0	2.9	0.0	0.0	0.0	0.0	0.0	0.0
Other antibiotic	0.9	1.5	2.8	0.1	0.3	0.0	1.9	0.6

Abbreviations: OM, otitis media; URI, upper respiratory infection.

[a] Grey box denotes appropriate treatment per guidelines. Antibiotic agents used by ≥ 1% of ≥ 1 infection cohorts are reported individually for all infection cohorts.

© 2022 Butler AM et al. *JAMA Network Open.*

eTable 10. Additional Selected Baseline Characteristics of Children Diagnosed with Infections of Interest (N = 2,804,245)[a,b]

	**Bacterial Infections[b] (Primary Analysis)**		**Viral Infections[c] (Secondary Analysis)**	
	**Appropriate Antibiotic (n = 1,068,417)** n (%)	**Inappropriate Antibiotic (n = 532,602)** n (%)	**Appropriate Antibiotic (n = 977,102)** n (%)	**Inappropriate Antibiotic (n = 226,124)** n (%)
Month of index date				
January	89,025 (8.3)	41,551 (7.8)	128,353 (13.1)	20,665 (9.1)
February	95,772 (9.0)	44,518 (8.4)	138,248 (14.2)	21,144 (9.4)
March	81,209 (7.6)	37,208 (7.0)	77,932 (8.0)	15,483 (6.9)
April	99,330 (9.3)	47,990 (9.0)	71,800 (7.4)	18,203 (8.1)
May	96,124 (9.0)	47,500 (8.9)	57,026 (5.8)	16,120 (7.1)
June	65,590 (6.1)	32,487 (6.1)	38,941 (4.0)	11,839 (5.2)
July	60,403 (5.7)	31,389 (5.9)	37,395 (3.8)	11,680 (5.2)
August	77,803 (7.3)	43,478 (8.2)	58,276 (6.0)	16,137 (7.1)
September	102,873 (9.6)	55,176 (10.4)	86,661 (8.9)	22,270 (9.9)
October	79,887 (7.5)	41,495 (7.8)	72,242 (7.4)	18,219 (8.1)
November	103,231 (9.7)	52,361 (9.8)	93,427 (9.6)	25,436 (11.3)
December	117,170 (11.0)	57,449 (10.8)	116,801 (12.0)	28,928 (12.8)
Year of index date				
2016	355,610 (33.3)	192,671 (36.2)	273,555 (28.0)	79,802 (35.3)
2017	453,654 (42.5)	222,331 (41.7)	417,590 (42.7)	95,774 (42.4)
2018	259,153 (24.3)	117,600 (22.1)	285,957 (29.3)	50,548 (22.4)
Baseline Elixhauser Comorbidities				
Alcohol abuse	232 (0.02)	172 (0.03)	193 (0.02)	71 (0.03)
Deficiency anemias	814 (0.1)	316 (0.1)	899 (0.1)	147 (0.1)
Rheumatoid arthritis/ collagen vascular disease	193 (0.0)	160 (0.0)	164 (0.0)	51 (0.0)
Chronic blood loss anemia	22 (0.0)	16 (0.0)	13 (0.0)	1 (0.0)
Chronic pulmonary disease	11,916 (1.1)	7,693 (1.4)	13,886 (1.4)	3,004 (1.3)
Congestive heart failure	43 (0.0)	8 (0.0)	59 (0.0)	8 (0.0)
Kidney failure	34 (0.0)	12 (0.0)	18 (0.0)	11 (0.0)

© 2022 Butler AM et al. *JAMA Network Open.*

	Bacterial Infections[b] (Primary Analysis)		Viral Infections[c] (Secondary Analysis)	
	**Appropriate Antibiotic (n = 1,068,417)** n (%)	**Inappropriate Antibiotic (n = 532,602)** n (%)	**Appropriate Antibiotic (n = 977,102)** n (%)	**Inappropriate Antibiotic (n = 226,124)** n (%)
Coagulopathy	103 (0.0)	78 (0.0)	130 (0.0)	27 (0.0)
Depression	6,982 (0.7)	5,042 (1.0)	5,963 (0.6)	1,799 (0.8)
Diabetes (complicated and uncomplicated)	1,915 (0.2)	1,481 (0.3)	1,568 (0.2)	533 (0.2)
Drug abuse	568 (0.1)	420 (0.1)	535 (0.1)	201 (0.1)
Hypertension	239 (0.0)	191 (0.0)	252 (0.0)	71 (0.0)
Hypothyroidism	559 (0.1)	406 (0.1)	605 (0.1)	176 (0.1)
Liver disease	50 (0.0)	42 (0.0)	56 (0.0)	16 (0.0)
Fluid and electrolyte disorders	519 (0.1)	263 (0.1)	477 (0.1)	100 (0.0)
Other neurological disorders	2,580 (0.2)	1,362 (0.3)	2,537 (0.3)	556 (0.3)
Obesity	20,496 (1.9)	12,475 (2.3)	18,202 (1.9)	4,540 (2.0)
Paralysis	1,006 (0.1)	481 (0.1)	1,009 (0.1)	234 (0.1)
Peripheral vascular disorders	14 (0.0)	16 (0.0)	12 (0.0)	3 (0.0)
Pulmonary circulation disorders	2,369 (0.2)	1,607 (0.3)	1,884 (0.2)	574 (0.3)
Psychoses	26 (0.0)	9 (0.0)	45 (0.0)	5 (0.0)
Valvular disease	255 (0.0)	124 (0.0)	320 (0.0)	64 (0.0)
Weight loss	4,259 (0.4)	1,814 (0.3)	5,345 (0.6)	854 (0.4)
Prior durable medical equipment				
Oxygen equipment	290 (0.0)	133 (0.0)	490 (0.1)	71 (0.0)
Home hospital bed	9 (0.0)	11 (0.0)	18 (0.0)	3 (0.0)
Mobility aids	472 (0.0)	276 (0.1)	426 (0.0)	121 (0.1)
Prior other frailty markers				
Ambulance / life support	3,996 (0.4)	2,113 (0.4)	4,474 (0.5)	811 (0.4)
Non-SNF-related rehabilitation services	41,406 (3.9)	23,806 (4.5)	39,614 (4.1)	8,722 (3.9)

Abbreviations: SNF, skilled nursing facility.

© 2022 Butler AM et al. *JAMA Network Open.*

[a] Elixhauser comorbidities, durable medical equipment, and other frailty markers were assessed from days -180 to 0 from the index date.
[b] For children diagnosed with bacterial infections (i.e., suppurative OM, pharyngitis, or sinusitis), antibiotic prescriptions were was categorized as appropriate (i.e., first-line antibiotic agent) or inappropriate (i.e., non-first-line antibiotic agent); patients without an antibiotic prescription were excluded. First-line antibiotic agents were defined as amoxicillin for suppurative OM; amoxicillin or penicillin for pharyngitis; and amoxicillin or amoxicillin-clavulanate for sinusitis.
[c] For children diagnosed with viral infections (i.e., influenza, viral URI, bronchiolitis, bronchitis, or non-suppurative OM,), antibiotic prescriptions were categorized as appropriate (no antibiotic) or inappropriate (antibiotic).

© 2022 Butler AM et al. *JAMA Network Open.*

eTable 11. Number of Exclusions For Adverse Drug Event Outcomes That Occurred Within 30 Days Prior to the Index Date

Adverse Drug Event Outcome	Bacterial Infections			Viral Infections				
	Suppurative OM N=601,711 n (%)	Pharyngitis N=617,215 n (%)	Sinusitis N=382,093 n (%)	Influenza N=180,996 n (%)	Viral URI N=772,040 n (%)	Bronchiolitis N=23,931 n (%)	Bronchitis N=72,407 n (%)	Nonsuppurative OM N=153,852 n (%)
Nausea / vomiting / abdominal pain	5,004 (0.8)	4,379 (0.7)	3,635 (1.0)	1,700 (0.9)	5,000 (0.6)	286 (1.2)	612 (0.8)	1,087 (0.7)
Non-*C. difficile* diarrhea	5,039 (0.8)	3,456 (0.6)	2,281 (0.6)	1,152 (0.6)	7,429 (1.0)	435 (1.8)	363 (0.5)	1,262 (0.8)
C. difficile infection	0 (0.0)	1 (0.0)	2 (0.0)	0 (0.0)	1 (0.0)	0 (0.0)	1 (0.0)	1 (0.0)
Anaphylaxis / angioedema / laryngeal edema	70 (0.0)	69 (0.0)	59 (0.0)	21 (0.0)	121 (0.0)	2 (0.0)	9 (0.0)	24 (0.0)
Skin rash / urticaria	4,847 (0.8)	6,614 (1.1)	2,089 (0.5)	603 (0.3)	8,330 (1.1)	290 (1.2)	286 (0.4)	1,291 (0.8)
Unspecified allergy	348 (0.1)	336 (0.1)	301 (0.1)	64 (0.0)	970 (0.1)	26 (0.1)	72 (0.1)	119 (0.1)
Tendinopathy	237 (0.0)	479 (0.1)	441 (0.1)	90 (0.0)	482 (0.1)	0 (0.0)	82 (0.1)	66 (0.0)
Stevens-Johnson syndrome / toxic epidermal necrolysis	0 (0.0)	0 (0.0)	1 (0.0)	0 (0.0)	2 (0.0)	0 (0.0)	1 (0.0)	1 (0.0)
Acute kidney failure	5 (0.0)	2 (0.0)	3 (0.0)	2 (0.0)	2 (0.0)	0 (0.0)	0 (0.0)	1 (0.0)

Abbreviations: OM, otitis media; URI, upper respiratory infection.

© 2022 Butler AM et al. *JAMA Network Open.*

eTable 12. Unadjusted and Propensity Score-weighted Hazard Ratio Estimates of Adverse Drug Events Following Inappropriate versus Appropriate Antibiotic Prescriptions Among Pediatric Patients

Index Diagnosis / Adverse Drug Event Outcome	HR (95% CI)[a]	
	Unadjusted	IPT-weighted[b]
Bacterial infections (primary analysis)		
Suppurative OM		
Nausea / vomiting / abdominal pain	1.17 (1.09-1.27)	1.20 (1.10-1.30)
Non-*C. difficile* diarrhea	1.00 (0.93-1.08)	1.30 (1.20-1.41)
C. difficile infection	5.78 (2.11-15.82)	6.23 (2.24-17.32)
Anaphylaxis / angioedema / laryngeal edema	3.25 (1.97-5.35)	4.14 (2.48-6.92)
Skin rash / urticaria	0.47 (0.44-0.50)	0.62 (0.58-0.66)
Unspecified allergy	0.58 (0.50-0.67)	0.67 (0.57-0.78)
Tendinopathy	1.34 (1.09-1.64)	0.91 (0.74-1.12)
Stevens-Johnson syndrome / toxic epidermal necrolysis	NE	NE
Acute kidney failure	NE	NE
Pharyngitis		
Nausea / vomiting / abdominal pain	1.23 (1.15-1.32)	1.20 (1.12-1.28)
Non-*C. difficile* diarrhea	1.13 (1.03-1.23)	1.16 (1.06-1.27)
C. difficile infection	6.60 (2.44-17.88)	8.42 (3.09-22.95)
Anaphylaxis / angioedema / laryngeal edema	1.41 (0.88-2.29)	1.49 (0.91-2.43)
Skin rash / urticaria	0.52 (0.49-0.56)	0.60 (0.56-0.65)
Unspecified allergy	0.63 (0.54-0.74)	0.69 (0.59-0.81)
Tendinopathy	1.58 (1.37-1.83)	1.25 (1.08-1.45)
Stevens-Johnson syndrome / toxic epidermal necrolysis	NE	NE
Acute kidney failure	NE	NE
Sinusitis		
Nausea / vomiting / abdominal pain	1.12 (1.03-1.22)	1.06 (0.97-1.16)
Non-*C. difficile* diarrhea	1.01 (0.82-1.12)	1.11 (1.00-1.23)
C. difficile infection	2.57 (0.92-7.27)	2.99 (1.05-8.49)
Anaphylaxis / angioedema / laryngeal edema	0.69 (0.40-1.21)	0.71 (0.39-1.28)
Skin rash / urticaria	0.56 (0.50-0.62)	0.64 (0.58-0.71)
Unspecified allergy	0.70 (0.56-0.89)	0.74 (0.58-0.93)
Tendinopathy	1.24 (1.06-1.44)	1.07 (0.91-1.25)
Stevens-Johnson syndrome / toxic epidermal necrolysis	NE	NE
Acute kidney failure	NE	NE
Viral infections (secondary analysis)		
Influenza		
Nausea / vomiting / abdominal pain	1.06 (0.78-1.44)	0.98 (0.70-1.37)
Non-*C. difficile* diarrhea	0.70 (0.43-1.15)	0.55 (0.31-0.95)
C. difficile infection	NE	NE
Anaphylaxis / angioedema / laryngeal edema	NE	NE
Skin rash / urticaria	1.58 (1.02-2.45)	1.30 (0.81-2.10)

© 2022 Butler AM et al. *JAMA Network Open.*

Index Diagnosis / Adverse Drug Event Outcome	HR (95% CI)[a]	
	Unadjusted	IPT-weighted[b]
Unspecified allergy	NE	NE
Tendinopathy	0.83 (0.27-1.88)	0.93 (0.39-2.20)
Stevens-Johnson syndrome / toxic epidermal necrolysis	NE	NE
Acute kidney failure	NE	NE
Viral URI		
Nausea / vomiting / abdominal pain	0.99 (0.89-1.09)	0.97 (0.86-1.09)
Non-*C. difficile* diarrhea	0.93 (0.83-1.03)	1.12 (0.98-1.27)
C. difficile infection	NE	NE
Anaphylaxis / angioedema / laryngeal edema	0.69 (0.43-1.12)	0.76 (0.45-1.28)
Skin rash / urticaria	1.79 (1.62-1.99)	2.35 (2.09-2.65)
Unspecified allergy	2.25 (1.77-2.85)	2.77 (2.09-3.67)
Tendinopathy	1.16 (0.93-1.43)	0.98 (0.76-1.27)
Stevens-Johnson syndrome / toxic epidermal necrolysis	NE	NE
Acute kidney failure	NE	NE
Bronchiolitis		
Nausea / vomiting / abdominal pain	0.81 (0.44-1.50)	1.61 (0.68-3.81)
Non-*C. difficile* diarrhea	1.29 (0.85-1.96)	1.68 (0.99-2.84)
C. difficile infection	NE	NE
Anaphylaxis / angioedema / laryngeal edema	NE	NE
Skin rash / urticaria	1.33 (0.71-2.51)	1.46 (0.71-2.99)
Unspecified allergy	NE	NE
Tendinopathy	NE	NE
Stevens-Johnson syndrome / toxic epidermal necrolysis	NE	NE
Acute kidney failure	NE	NE
Bronchitis		
Nausea / vomiting / abdominal pain	0.68 (0.55-0.85)	0.72 (0.57-0.90)
Non-*C. difficile* diarrhea	0.86 (0.63-1.19)	0.82 (0.58-1.14)
C. difficile infection	NE	NE
Anaphylaxis / angioedema / laryngeal edema	NE	NE
Skin rash / urticaria	1.24 (0.87-1.76)	1.20 (0.83-1.73)
Unspecified allergy	1.19 (0.64-2.22)	1.47 (0.75-2.87)
Tendinopathy	0.89 (0.59-1.35)	0.77 (0.49-1.19)
Stevens-Johnson syndrome / toxic epidermal necrolysis	NE	NE
Acute kidney failure	NE	NE
Non-suppurative OM		
Nausea / vomiting / abdominal pain	0.86 (0.73-1.01)	0.84 (0.69-1.01)
Non-*C. difficile* diarrhea	1.25 (1.08-1.44)	1.19 (1.01-1.40)
C. difficile infection	NE	NE
Anaphylaxis / angioedema / laryngeal edema	0.56 (0.21-1.52)	0.52 (0.19-1.43)
Skin rash / urticaria	2.55 (2.23-2.93)	2.21 (1.91-2.56)
Unspecified allergy	3.23 (2.26-4.62)	3.18 (2.13-4.77)

© 2022 Butler AM et al. *JAMA Network Open.*

Index Diagnosis / Adverse Drug Event Outcome	HR (95% CI)[a]	
	**Unadjusted**	**IPT-weighted[b]**
Tendinopathy	0.75 (0.50-1.11)	0.73 (0.49-1.10)
Stevens-Johnson syndrome / toxic epidermal necrolysis	NE	NE
Acute kidney failure	NE	NE

Abbreviations: CI, confidence interval; HR, hazard ratio; IPT, inverse probability of treatment; NE, not estimable; OM, otitis media; URI, upper respiratory infection.

[a] For hazard ratio estimation, we required ≥ 5 adverse event cases in both the reference category (i.e., appropriate antibiotic prescription) and the comparator group (i.e., inappropriate antibiotic prescription) to ensure stability of the effect estimate.
[b] Results for weighted hazard ratios are also presented in Figure 1 of main manuscript.

© 2022 Butler AM et al. *JAMA Network Open.*

eTable 13. Inverse Probability Of Treatment–Weighted 30-Day All Cause and Adverse Drug Event–Related Attributable Expenditure Estimates of Inappropriate Antibiotic Prescriptions Among Children by Setting[a]

Expenditure Category	Patient-Level Attributable Expenditure Estimates (95% CI), $
Bacterial infections (primary analysis)	
Suppurative OM	
All-cause total	56 (43, 68)
Inpatient medical	3 (-9, 14)
Emergency department	2 (0, 3)
Outpatient medical	17 (13, 22)
Outpatient pharmacy	34 (30, 38)
Adverse drug event-related	2 (0, 5)
Pharyngitis	
All-cause total	42 (29, 52)
Inpatient medical	-1 (-12, 9)
Emergency department	5 (3, 7)
Outpatient medical	13 (10, 17)
Outpatient pharmacy	25 (22, 28)
Adverse drug event-related	1 (-4, 6)
Sinusitis	
All-cause total	21 (3, 36)
Inpatient medical	-1 (-16, 12)
Emergency department	1 (-2, 4)
Outpatient medical	1 (-7, 8)
Outpatient pharmacy	20 (16, 24)
Adverse drug event-related	0 (-8, 7)
Viral infections (secondary analysis)	
Influenza	
All-cause total	97 (43, 141)
Inpatient medical	-6 (-49, 28)
Emergency department	-3 (-19, 16)
Outpatient medical	69 (41, 86)
Outpatient pharmacy	38 (29, 47)
Adverse drug event-related	19 (-30, 40)
Viral URI	
All-cause total	81 (54, 106)
Inpatient medical	23 (-5, 44)
Emergency department	2 (-2, 6)
Outpatient medical	35 (28, 43)
Outpatient pharmacy	21 (17, 25)
Adverse drug event-related	11 (1, 17)
Bronchiolitis	
All-cause total	-8 (-98, 67)
Inpatient medical	-74 (-156, -18)
Emergency department	11 (-32, 47)

© 2022 Butler AM et al. *JAMA Network Open.*

Expenditure Category	Patient-Level Attributable Expenditure Estimates (95% CI), $
Outpatient medical	20 (-36, 57)
Outpatient pharmacy	35 (17, 48)
Adverse drug event-related	56 (-61, 109)
Bronchitis	
All-cause total	-24 (-56, 11)
Inpatient medical	8 (-13, 33)
Emergency department	-19 (-27, -10)
Outpatient medical	-34 (-50, -17)
Outpatient pharmacy	22 (12, 31)
Adverse drug event-related	-10 (-22, 7)
Non-suppurative OM	
All-cause total	-96 (-124, -73)
Inpatient medical	0 (-21, 19)
Emergency department	-6 (-11, -2)
Outpatient medical	-112 (-124, -101)
Outpatient pharmacy	22 (15, 28)
Adverse drug event-related	3 (-1, 7)

Abbreviations: CI, confidence interval; OM, otitis media; URI, upper respiratory infection

[a] In the inverse probability of treatment (IPT)-weighted population, all measured baseline characteristics were well-balanced between treatment groups (standardized mean differences [SMD] < 0.10) with the following exceptions: provider specialty for all-cause and ADE-related expenditures for the suppurative otitis media (SMD=0.14), influenza (SMD=0.14), and bronchiolitis (SMD=0.18) cohorts.

© 2022 Butler AM et al. *JAMA Network Open.*

eTable 14. Inverse Probability of Treatment–Weighted 30-Day Adverse Drug Event–Related Health Care Utilization and Total Per-Patient Expenditure Estimates of Inappropriate Antibiotic Prescriptions Among Children

	Appropriate Antibiotic	Inappropriate Antibiotic	Appropriate Antibiotic	Inappropriate Antibiotic
**Index diagnosis**	**Health Care Utilization N (%)**	**Health Care Utilization N (%)**	**Total Per-patient Expenditure Estimates (mean (SD)), $**	**Total Per-patient Expenditure Estimates (mean (SD)), $**
Bacterial infections (primary analysis)				
Suppurative OM	9,424 (2.3)	3,756 (2.1)	16 (422)	19 (758)
Pharyngitis	7,956 (2.0)	3,578 (1.8)	19 (843)	22 (1,377)
Sinusitis	4,211 (1.8)	2,100 (1.6)	21 (849)	23 (1,653)
Viral infections (secondary analysis)				
Influenza	1,919 (1.2)	70 (1.1)	14 (450)	51 (2,640)
Viral URI	8,070 (1.3)	1,571 (1.8)	14 (520)	22 (992)
Bronchiolitis	366 (1.9)	72 (3.6)	16 (302)	49 (1,423)
Bronchitis	228 (1.1)	545 (1.1)	27 (973)	15 (376)
Non-suppurative OM	873 (1.4)	1,345 (2.2)	14 (436)	18 (426)

Abbreviations: OM, otitis media; SD, standard deviation; URI, upper respiratory infection.

© 2022 Butler AM et al. *JAMA Network Open.*

eTable 15. Total 30-Day Attributable Expenditure Estimates of Inappropriate Antibiotic Prescriptions in 2017 Pediatric MarketScan Study Population, Age 6 Months to 17 Years[a]

	Attributable Expenditures (2018 USD)				
	**Inpatient Medical, $**	**Emergency Department, $**	**Outpatient Medical, $**	**Outpatient Pharmacy, $**	**Total, $**
Bacterial infections (primary analysis)					
Suppurative OM	205,120 (-686,389, 1,023,786)	129,169 (-14,819, 258,000)	1,302,841 (975,856, 1,638,577)	2,564,018 (2,272,945, 2,818,236)	4,201,050 (3,248,039, 5,062,570)
Pharyngitis	-125,048 (-1,056,613, 743,823)	427,498 (231,817, 589,766)	1,117,876 (820,783, 1,473,947)	2,125,705 (1,888,669, 2,407,574)	3,545,682 (2,477,750, 4,425,400)
Sinusitis	-83,192 (-876,691, 660,927)	45,862 (-120,000, 208,550)	67,266 (-366,076, 452,932)	1,139,120 (920,392, 1,317,809)	1,168,699 (140,731, 1,995,259)
Viral infections (secondary analysis)					
Influenza	-17,600 (-142,670, 82,028)	-9,599 (-54,295, 45,260)	201,688 (119,337, 250,992)	109,680 (84,884, 137,385)	284,023 (125,835, 412,514)
Viral URI	894,350 (-194,798, 1,690,734)	72,385 (-73,884, 216,700)	1,357,454 (1,079,019, 1,653,214)	827,237 (649,309, 972,339)	3,150,638 (2,092,262, 4,119,284)
Bronchiolitis	-68,912 (-146,056, -16,668)	10,093 (-30,183, 43,534)	18,313 (-33,713, 53,201)	32,767 (16,237, 45,136)	-7,803 (-91,717, 62,651)
Bronchitis	164,411 (-263,597, 688,161)	-402,590 (-562,727, -210,225)	-717,702 (-1,032,880, -359,228)	463,832 (259,280, 641,196)	-492,599 (-1,167,722, 228,498)
Non-suppurative OM	-2,729 (-555,725, 505,091)	-161,490 (-285,601, -57,967)	-3,015,469 (-3,336,334, -2,709,938)	598,548 (413,101, 764,408)	-2,581,946 (-3,321,732, -1,954,618)

Abbreviations: OM, otitis media; URI, upper respiratory infection; USA, United States dollar.

[a] Bronchiolitis cohort was restricted to ages 6 months to 3 years; bronchitis cohort was restricted to ages 5–17 years.

© 2022 Butler AM et al. *JAMA Network Open.*

eTable 16. Confidence Intervals for Annual National Attributable 30-Day Expenditures of Inappropriate Antibiotic Prescriptions Among the US Commercially Insured Population, Age 6 Months to 17 Years[a]

	Attributable Expenditures (2018 USD)				
	**Inpatient Medical, $**	**Emergency Department, $**	**Outpatient Medical, $**	**Outpatient Pharmacy, $**	**Total, $**
Bacterial infections (primary analysis)					
Suppurative OM	1,235,313 (-4,133,695, 6,165,627)	777,904 (-89,245, 1,553,777)	7,846,200 (5,876,975, 9,868,131)	15,441,487 (13,688,535, 16,972,489)	25,300,317 (19,560,922, 30,488,716)
Pharyngitis	-750,188 (-6,338,857, 4,462,360)	2,564,653 (1,390,722, 3,538,139)	6,706,388 (4,924,062, 8,842,534)	12,752,577 (11,330,547, 14,443,571)	21,271,338 (14,864,574, 26,548,959)
Sinusitis	-503,873 (-5,309,896, 4,003,067)	277,773 (-726,807, 1,263,137)	407,416 (-2,217,231, 2,743,293)	6,899,358 (5,574,584, 7,981,632)	7,078,513 (852,372, 12,084,775)
Viral infections (secondary analysis)					
Influenza	-98,806 (-800,965, 460,513)	-53,888 (-304,819, 254,097)	1,132,300 (669,970, 1,409,102)	615,754 (476,547, 771,296)	1,594,541 (706,451, 2,315,904)
Viral URI	5,430,897 (-1,182,902, 10,266,901)	439,555 (-448,658, 1,315,898)	8,243,074 (6,552,289, 10,039,062)	5,023,360 (3,942,899, 5,904,483)	19,132,099 (12,705,163, 25,014,159)
Bronchiolitis	-334,451 (-708,855, -80,897)	48,984 (-146,488, 211,286)	88,877 (-163,622, 258,203)	159,028 (78,805, 219,061)	-37,871 (-445,134, 304,064)
Bronchitis	1,059,296 (-1,698,346, 4,433,790)	-2,593,873 (-3,625,627, -1,354,471)	-4,624,124 (-6,654,804, -2,314,488)	2,988,452 (1,670,530, 4,131,198)	-3,173,797 (-7,523,579, 1,472,204)
Non-suppurative OM	-16,270 (-3,313,681, 3,011,763)	-962,935 (-1,702,981, -345,646)	-17,980,659 (-19,893,913, -16,158,840)	3,569,023 (2,463,241, 4,558,019)	-15,395,644 (-19,806,845, -11,655,009)

Abbreviations: OM, otitis media; URI, upper respiratory infection; USA, United States dollar.

[a] Bronchiolitis cohort was restricted to ages 6 months to 3 years; bronchitis cohort was restricted to ages 5–17 years.

© 2022 Butler AM et al. *JAMA Network Open.*

eTable 17. Baseline Characteristics of Children Diagnosed with a Noninfectious Clinical Condition[a,b]

	Noninfectious Clinical Condition[c]	
	**Appropriate Antibiotic n = 546,793 (%)**	**Inappropriate Antibiotic n = 29,558 (%)**
Demographic characteristics		
Age, mean (SD), y	10 (3)	9 (5)
Male sex	310,579 (56.8)	16,754 (56.7)
Health insurance plan type		
Basic, comprehensive	79,808 (14.6)	3,983 (13.5)
CDHP	69,934 (12.8)	3,935 (13.3)
EPO or PPO	292,977 (53.6)	16,463 (55.7)
HMO	56,749 (10.4)	2,687 (55.7)
POS or POS with capitation	36,551 (6.7)	1,921 (6.5)
Unknown	10,774 (1.2)	569 (1.9)
Urban residence	464,023 (84.9)	24,134 (81.7)
Geographic region		
Midwest	110,739 (20.3)	4,671 (15.8)
Northeast	108,377 (19.8)	5,516 (18.9)
South	233,764 (42.8)	14,833 (50.2)
West	93,913 (17.2)	4,538 (15.4)
Month of index date		
January	23,760 (4.4)	1,843 (6.2)
February	25,067 (4.6)	2,020 (6.8)
March	30,610 (5.6)	1,976 (6.7)
April	56,028 (10.3)	3,076 (10.4)
May	59,906 (11.0)	2,914 (9.9)
June	46,950 (8.6)	1,797 (6.1)
July	50,393 (9.2)	1,437 (4.9)
August	72,439 (13.3)	2,345 (7.9)
September	64,596 (11.8)	3,530 (11.9)
October	45,299 (8.3)	2,665 (9.0)
November	40,170 (7.4)	3,078 (10.4)
December	31,575 (5.8)	2,877 (9.7)
Year of index date		
2016	204,683 (37.4)	11,320 (38.3)
2017	207,774 (38.0)	11,932 (40.4)
2018	134,336 (24.6)	6,306 (21.3)
Index characteristics		
Index provider specialty		
Emergency medicine	11,095 (2.0)	399 (1.4)
Internal medicine	12,494 (2.3)	787 (2.7)
Other/unknown	189,016 (34.6)	6,919 (23.4)
Pediatrics/family medicine	334,188 (61.1)	21,453 (72.6)
Index provider location		
Emergency department	22,941 (4.2)	294 (1.0)
Office	493,765 (90.3)	27,398 (92.7)
Other / unknown	18,467 (3.4)	576 (2.0)
Retail clinic	298 (0.1)	19 (0.1)
Urgent care center	11,322 (2.1)	1,271 (4.3)
Baseline characteristics prior to index		
Prior emergency department visit	36,476 (6.7)	2,151 (7.3)

© 2022 Butler AM et al. *JAMA Network Open.*

	Noninfectious Clinical Condition[c]	
	**Appropriate Antibiotic n = 546,793 (%)**	**Inappropriate Antibiotic n = 29,558 (%)**
Prior number of office visits, median (IQR)	1 (0-2)	1 (0-3)
Prior number of unique medication classes, median (IQR)	0 (0-1)	1 (0-2)
Baseline Elixhauser Comorbidities		
Alcohol abuse	150 (0.0)	8 (0.0)
Deficiency anemias	376 (0.1)	15 (0.1)
Rheumatoid arthritis/ collagen vascular disease	116 (0.0)	9 (0.0)
Chronic blood loss anemia	21 (0.0)	0 (0.0)
Chronic pulmonary disease	19,792 (3.6)	4,018 (13.6)
Congestive heart failure	7 (0.0)	1 (0.0)
Kidney failure	15 (0.0)	0 (0.0)
Coagulopathy	78 (0.0)	6 (0.0)
Depression	5,415 (1.0)	238 (0.8)
Diabetes (complicated and uncomplicated)	943 (0.2)	57 (0.2)
Drug abuse	445 (0.1)	35 (0.1)
Hypertension	225 (0.0)	14 (0.1)
Hypothyroidism	365 (0.1)	24 (0.1)
Liver disease	25 (0.0)	1 (0.0)
Fluid and electrolyte disorders	184 (0.0)	19 (0.1)
Other neurological disorders	1,475 (0.3)	83 (0.3)
Obesity	19,261 (3.5)	872 (3.0)
Paralysis	598 (0.1)	33 (0.1)
Peripheral vascular disorders	9 (0.0)	0 (0.0)
Pulmonary circulation disorders	20 (0.0)	0 (0.0)
Psychoses	1,669 (0.3)	82 (0.3)
Valvular disease	118 (0.0)	4 (0.0)
Weight loss	2,666 (0.5)	130 (0.4)
Prior durable medical equipment		
Oxygen equipment	253 (0.1)	13 (0.0)
Home hospital bed	11 (0.0)	2 (0.0)
Mobility aids	286 (0.1)	20 (0.1)
Prior other frailty markers		
Ambulance / life support	3,516 (0.6)	123 (0.4)
Non-SNF-related rehabilitation services	22,434 (4.1)	1,224 (4.1)

Abbreviations: CDHP, consumer directed health plan; EPO, exclusive provider organization; HMO, health maintenance organization; IQR, interquartile range; POS, point of service; PPO, preferred provider organization; SD, standard deviation; SNF, skilled nursing facility.

[a] Demographic characteristics were assessed on the index date. Index characteristics were assessed on the index date. Baseline characteristics prior to index were assessed in the 180-day baseline period before the index date (days -180 to -1) with the exception of Elixhauser comorbidities, durable medical equipment, and other frailty markers, which were assessed through the index date (days -180 to 0).
[b] Results are expressed as N (%) unless otherwise indicated.

© 2022 Butler AM et al. *JAMA Network Open.*

[c] For children diagnosed with asthma/allergy or asthma exacerbation, antibiotic prescriptions were categorized as appropriate (no antibiotic) or inappropriate (antibiotic).

© 2022 Butler AM et al. *JAMA Network Open.*

eTable 18. Distribution of Index Antibiotic Agents Prescribed to Children by Noninfectious Clinical Condition[a]

Antibiotic Agent	Asthma/Allergy n = 507,429 (%)	Asthma Exacerbation n = 68,922 (%)
None	96.7	81.2
Amoxicillin-clavulanate	0.3	2.2
Amoxicillin	1.0	6.6
Azithromycin	1.5	7.2
Cefdinir	0.3	2.0
Other antibiotic agent	0.3	0.9

[a] Grey box denotes appropriate treatment per guidelines. Antibiotic agents used by ≥ 1% of either noninfectious clinical condition cohort are reported individually for both cohorts.

© 2022 Butler AM et al. *JAMA Network Open.*

eTable 19. Number of Exclusions For Adverse Drug Event Outcomes That Occurred Within 30 Days Prior to The Index Date By Noninfectious Clinical Condition

	**Noninfectious Clinical Condition**	
**Adverse Drug Event Outcome**	**Asthma/Allergy N=507,429 (%)**	**Asthma Exacerbation N=68,922 (%)**
Nausea / vomiting / abdominal pain	3,388 (0.7)	1,510 (2.2)
Non-*C. difficile* diarrhea	2,800 (0.6)	434 (0.6)
C. difficile infection	4 (0.0)	2 (0.0)
Anaphylaxis / angioedema / laryngeal edema	180 (0.0)	13 (0.0)
Skin rash / urticaria	11,769 (2.3)	613 (0.9)
Unspecified allergy	16,828 (3.3)	170 (0.3)
Tendinopathy	567 (0.1)	69 (0.1)
Stevens-Johnson syndrome / toxic epidermal necrolysis	5 (0.0)	0 (0.0)
Acute kidney failure	6 (0.0)	1 (0.0)

© 2022 Butler AM et al. *JAMA Network Open.*

eTable 20. Unadjusted and Propensity Score–Weighted Hazard Ratio Estimates of Adverse Drug Events Following Inappropriate vs Appropriate Antibiotic Prescriptions Among Pediatric Patients by Noninfectious Clinical Condition

Index Diagnosis / Adverse Drug Event Outcome	HR (95% CI)[a]	
	Unadjusted	IPT-weighted[b]
Asthma/Allergy		
Nausea / vomiting / abdominal pain	1.18 (0.94–1.48)	1.09 (0.85-1.41)
Non-*C. difficile* diarrhea	1.14 (0.86–1.51)	1.03 (0.74-1.43)
C. difficile infection	NE	NE
Anaphylaxis / angioedema / laryngeal edema	0.44 (0.27-0.74)	0.92 (0.46-1.83)
Skin rash / urticaria	1.59 (1.25-2.02)	1.96 (1.44-2.66)
Unspecified allergy	1.96 (1.23-3.11)	2.77 (1.50-5.11)
Tendinopathy	0.57 (0.34-0.95)	0.45 (0.26-0.77)
Stevens-Johnson syndrome / toxic epidermal necrolysis	NE	NE
Acute kidney failure	NE	NE
Asthma Exacerbation		
Nausea / vomiting / abdominal pain	0.93 (0.72-1.22)	0.84 (0.72-1.23)
Non-*C. difficile* diarrhea	0.94 (0.69-1.30)	0.96 (0.69-1.34)
C. difficile infection	NE	NE
Anaphylaxis / angioedema / laryngeal edema	NE	NE
Skin rash / urticaria	1.55 (1.14-2.11)	1.55 (1.13-2.14)
Unspecified allergy	1.01 (0.52-1.97)	1.24 (0.57-2.71)
Tendinopathy	1.23 (0.75-2.05)	1.31 (0.76-2.24)
Stevens-Johnson syndrome / toxic epidermal necrolysis	NE	NE
Acute kidney failure	NE	NE

Abbreviations: CI, confidence interval; HR, hazard ratio;; IPT, inverse probability of treatment; NE, not estimable.

[a] For hazard ratio estimation, we required ≥ 5 adverse event cases in both the reference category (i.e., appropriate antibiotic prescription) and the comparator group (i.e., inappropriate antibiotic prescription) to ensure stability of the effect estimate.

© 2022 Butler AM et al. *JAMA Network Open.*

eTable 21. Inverse Probability of Treatment–Weighted 30-Day Health Care Utilization and All-Cause and Adverse Drug Event–Related Total Per-Patient and Attributable Expenditure Estimates of Inappropriate Antibiotic Prescriptions Among Children by Noninfectious Clinical Condition[a]

Expenditure Category	Appropriate Health Care Utilization N (%)	Inappropriate Health Care Utilization N (%)	Appropriate Total Per-patient Expenditure Estimates (mean (SD)), $	Inappropriate Total Per-patient Expenditure Estimates (mean (SD)), $	Patient-level Attributable Expenditure Estimates (95% CI), $
Asthma/Allergy					
All-cause total expenditures	465,736 (100.0)	15,834 (100.0)	727 (2,969)	928 (8,675)	246 (147, 327)
Inpatient medical expenditures	1,101 (0.2)	42 (0.3)	54 (2,354)	164 (8,401)	60 (-23, 122)
Emergency department expenditures	23,918 (5.1)	1,003 (6.3)	93 (626)	70 (559)	28 (11, 45)
Outpatient medical expenditures	457,914 (98.3)	15,435 (97.5)	455 (1,410)	506 (1,638)	95 (60, 126)
Outpatient pharmacy expenditures	250,147 (53.7)	15,833 (100.0)	125 (641)	188 (990)	64 (46, 77)
Adverse drug event-related expenditures	6,143 (1.3)	266 (1.7)	17 (1,762)	52 (2,722)	21 (-13, 45)
Asthma Exacerbation					
All-cause total expenditures	48,432 (100.0)	12,363 (100.0)	947 (4,131)	1019 (9,196)	48 (-96, 162)
Inpatient medical expenditures	206 (0.4)	45 (0.4)	102 (3,622)	170 (9,013)	50 (-110, 155)
Emergency department expenditures	1,459 (3.0)	319 (2.6)	73 (672)	66 (557)	-6 (-18, 7)
Outpatient medical expenditures	48,410 (100.0)	12,361 (100.0)	529 (1,314)	512 (1,418)	-20 (-48, 5)
Outpatient pharmacy expenditures	34,628 (71.5)	12,359 (100.0)	243 (1,018)	272 (726)	23 (9, 37)
Adverse drug event-related expenditures	667 (1.4)	197 (1.6)	50 (5,275)	30 (722)	-26 (-59, 46)

Abbreviations: CI, confidence interval; SD, standard deviation.

[a] In the inverse probability of treatment (IPT)-weighted population, all measured baseline characteristics were well-balanced between treatment groups (standardized mean differences [SMD] < 0.10) with the following exceptions: provider specialty for all-cause and ADE-related expenditures for the asthma/allergy cohort (SMD=0.11), and plan type for all-cause and ADE-related expenditures for the asthma/allergy cohort (SMD=0.15).

© 2022 Butler AM et al. *JAMA Network Open.*

eTable 22. Total Attributable Expenditures of Inappropriate Antibiotic Prescriptions Among Children by Noninfectious Clinical Condition

	Attributable Expenditures (2018 USD)				
**Index Diagnoses**	**Inpatient Medical, $**	**Emergency Department, $**	**Outpatient Medical, $**	**Outpatient Pharmacy, $**	**Total, $**
Noninfectious clinical condition- standardized to the 2017 US employer-insured population					
Asthma/Allergy	2,439,085 (-950,915, 4,960,713)	1,140,576 (435,878, 1,830,835)	3,848,991 (2,435,651, 5,136,757)	2,587,002 (1,876,808, 3,141,250)	10,013,746 (5,974,159, 13,298,088)
Asthma Exacerbation	1,641,822 (-3,589,834, 5,077,715)	-197,130 (-580,191, 236,145)	-645,578 (-1,583,992, 154,846)	765,364 (299,895, 1,211,160)	1,564,286 (-3,159,342, 5,290,930)
Noninfectious clinical condition- in 2017 MarketScan study population					
Asthma/Allergy	390,877 (-152,389, 794,981)	182,784 (69,852, 293,401)	616,822 (390,326, 823,193)	414,581 (300,769, 503,402)	1,604,758 (957,392, 2,131,091)
Asthma Exacerbation	256,737 (-561,353, 794,018)	-30,826 (-90,726, 36,927)	-100,951 (-247,694, 24,214)	119,682 (46,895, 189,393)	244,612 (-494,036, 827,359)

Abbreviations: USD, United States dollar..

© 2022 Butler AM et al. *JAMA Network Open.*

eTable 23. Sensitivity Analyses for Inverse Probability of Treatment–Weighted All-Cause Attributable Expenditure Estimates of Inappropriate Antibiotic Prescriptions Among Children by Condition

Index Diagnosis	Patient-level All-Cause Attributable Expenditure Estimates (95% CI), $
**90-day expenditures**	
Bacterial infections (primary analysis)	
Suppurative OM	105 (83, 129)
Pharyngitis	72 (43, 98)
Sinusitis	14 (-33, 57)
Viral infections (secondary analysis)	
Influenza	521 (-302, 979)
Viral URI	157 (106, 202)
Bronchiolitis	107 (-150, 324)
Bronchitis	-7 (-78, 74)
Non-suppurative OM	-114 (-171, -61)
**30-day expenditures, excluding HMO and POS with capitation plans**	
Bacterial infections (primary analysis)	
Suppurative OM	52 (41, 66)
Pharyngitis	41 (30, 51)
Sinusitis	24 (4, 43)
Viral infections (secondary analysis)	
Influenza	82 (24, 321)
Viral URI	79 (47, 103)
Bronchiolitis	16 (-91, 100)
Bronchitis	-20 (-56, 17)
Non-suppurative OM	-104 (-127, -79)
**30-day expenditures, inappropriate antibiotic definition expanded to include inappropriate agent or duration[a]**	
Bacterial infections (primary analysis)	
Suppurative OM	44 (12, 74)
Pharyngitis	39 (25, 51)
Sinusitis	18 (3, 36)

Abbreviations: CI, confidence interval; OM, otitis media; URI, upper respiratory infection.

[a] Inappropriate duration was only relevant for bacterial infections; any antibiotic was considered inappropriate for viral infections.

© 2022 Butler AM et al. *JAMA Network Open.*

eFigure 1. Derivation of Pediatric Infection Cohort in MarketScan Commercial Database (Index Events April 1, 2016, to September 30, 2018)

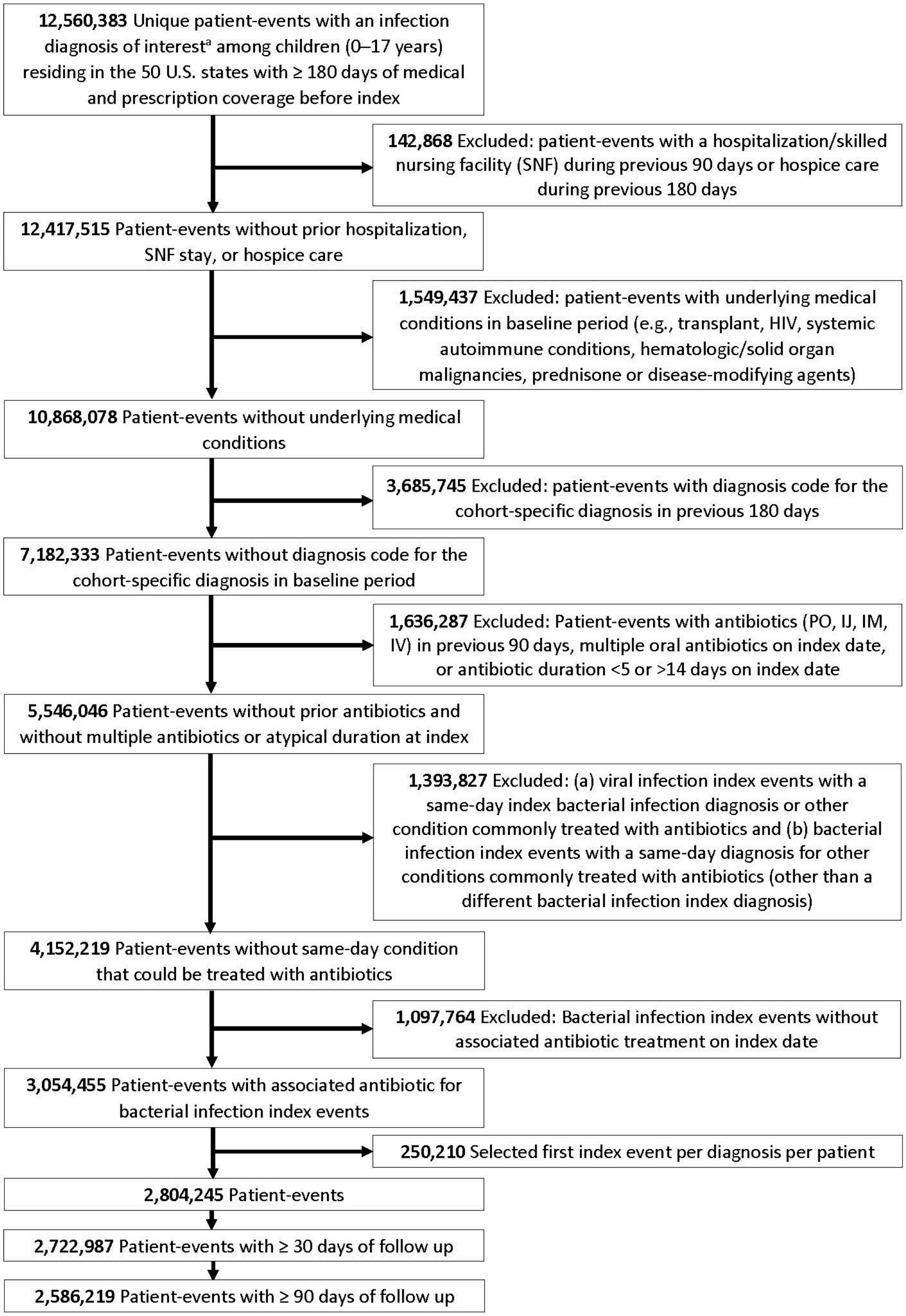

© 2022 Butler AM et al. *JAMA Network Open.*

[s] Infections of interest include three bacterial infections (suppurative OM, pharyngitis, and sinusitis) and five viral infections (influenza, viral URI, bronchiolitis, bronchitis, and non-suppurative OM) which were identified on outpatient claims with a claimline payment of > $0.

© 2022 Butler AM et al. *JAMA Network Open.*

eFigure 2. Standardized Mean Differences of Patient- and Provider-Level Characteristics Between Treatment Groups, in the Unweighted and Weighted Pediatric Populations, for Acute Kidney Failure Outcome Cohort[a,b,c,d,e]

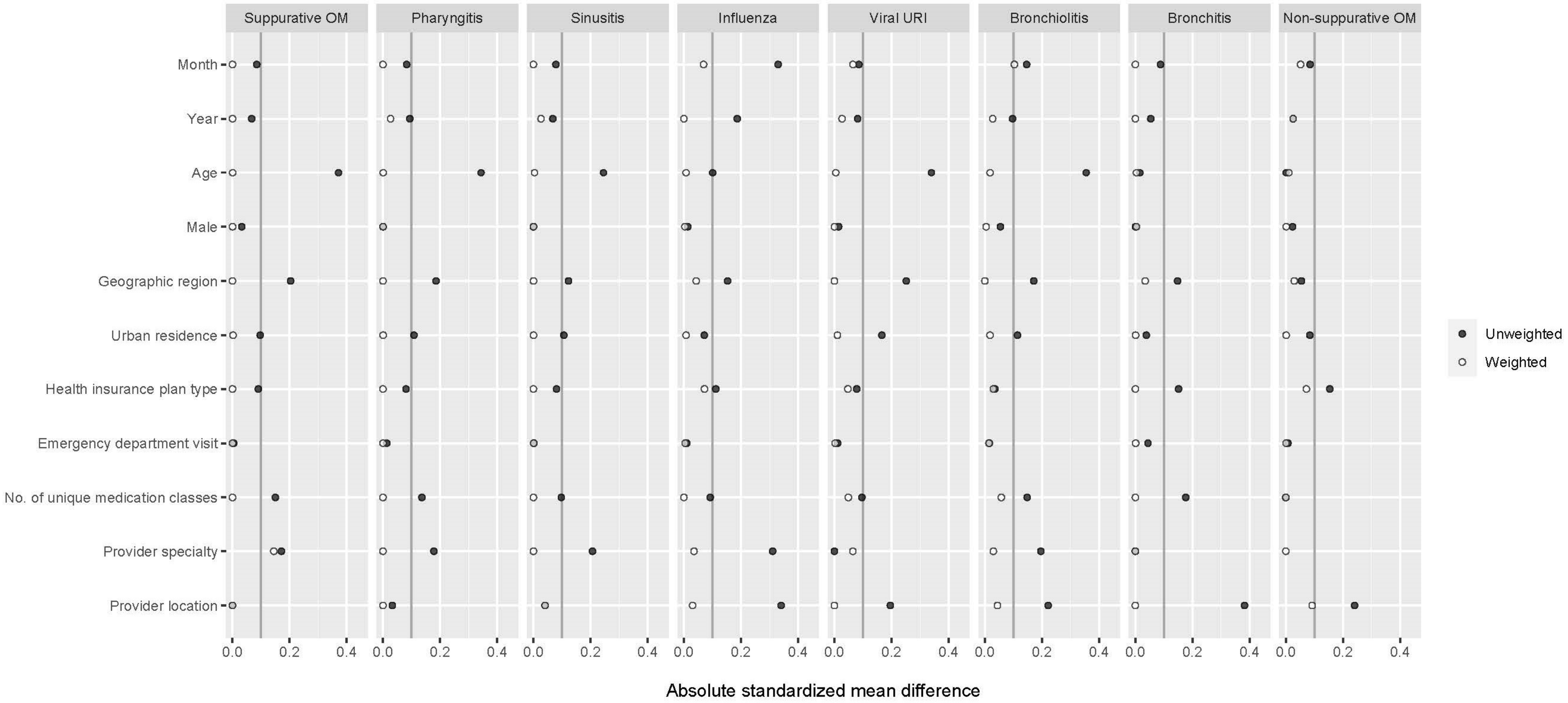

Abbreviations: OM, otitis media; URI, upper respiratory infection.

[a] Standardized mean difference (SMD) calculated as the difference in means or proportions of a variable divided by the pooled standard deviation of the variable. All standardized mean difference estimates compare children who received an appropriate antibiotic (reference) versus an inappropriate antibiotic within each infection cohort, after cohort-specific trimming. Standardized mean differences <0.1 indicate no substantial difference in means or proportions between groups. In the inverse probability of treatment (IPT)-weighted population, all measured baseline characteristics were well-balanced between treatment groups (standardized mean differences < 0.10) with the following exceptions: provider specialty for suppurative otitis media for the analysis of nausea/vomiting/abdominal pain, non-*C. difficile* diarrhea, *C. difficile* infection, anaphylaxis/angioedema/laryngeal edema, skin rash/urticaria, unspecified allergy, and tendinopathy (all SMDs=0.15); month of index for bronchiolitis for the analysis of nausea/vomiting/abdominal pain, non-*C. difficile* diarrhea, *C. difficile* infection, anaphylaxis/angioedema/laryngeal edema, unspecified allergy, and tendinopathy (all SMDs=0.10); month of index for viral URI for the analysis of nausea/vomiting/abdominal pain (SMD=0.11).

© 2022 Butler AM et al. *JAMA Network Open.*

[b] Each outcome was evaluated in a separate cohort with a separate propensity score model and weights. The acute kidney failure cohort is presented here as a representative example because it had few cases of acute kidney failure in the 30 days prior to the index date (i.e., 15 cases across all viral and bacterial infection cohorts) and thus made few exclusions based on previous ascertainment of the outcome.
[c] Demographics were identified in the month of index. Emergency department visits and unique medication classes were identified in the baseline period before index. Provider specialty and provider location refer to the index diagnosis.
[d] Grey shaded circles reflect overlap of unweighted and weighted SMDs.
[e] The unweighted SMD for provider specialty for the non-suppurative OM cohort is not presented as the SMD was >0.6.

© 2022 Butler AM et al. *JAMA Network Open.*

eFigure 3. Propensity Score–Weighted Hazard Ratio Estimates of Additional Adverse Drug Events Following Inappropriate vs Appropriate Antibiotic Prescriptions Among Pediatric Patients[a, b]

	No. of events (Rate per 10,000 person-days)			
	Appropriate Agent	Inappropriate Agent	Weighted HR (95% CI)	Inappropriate agent nonharmful / Inappropriate agent harmful
**Stevens-Johnson syndrome/toxic epidermal necrolysis**				
Suppurative OM	1 (0.00)	0 (0.00)	NE	
Pharyngitis	4 (0.01)	2 (0.01)	NE	
Sinusitis	0 (0.00)	1 (0.01)	NE	
Influenza	0 (0.00)	0 (0.00)	NE	
Viral URI	4 (0.00)	1 (0.01)	NE	
Bronchiolitis	0 (0.00)	0 (0.00)	NE	
Bronchitis	0 (0.00)	0 (0.00)	NE	
Non-suppurative OM	0 (0.00)	0 (0.00)	NE	
**Acute kidney failure**				
Suppurative OM	1 (0.00)	1 (0.00)	NE	
Pharyngitis	8 (0.01)	2 (0.01)	NE	
Sinusitis	4 (0.01)	1 (0.01)	NE	
Influenza	2 (0.01)	0 (0.00)	NE	
Viral URI	6 (0.01)	2 (0.02)	NE	
Bronchiolitis	0 (0.00)	0 (0.00)	NE	
Bronchitis	0 (0.00)	0 (0.00)	NE	
Non-suppurative OM	0 (0.00)	1 (0.01)	NE	
**Tendinopathy plus tendon rupture**				
Suppurative OM	254 (0.08)	151 (0.11)	0.91 (0.74, 1.12)	
Pharyngitis	413 (0.13)	328 (0.20)	1.25 (1.08, 1.45)	
Sinusitis	390 (0.21)	269 (0.26)	1.07 (0.91, 1.25)	
Influenza	178 (0.13)	6 (0.11)	0.93 (0.39, 2.20)	
Viral URI	582 (0.12)	98 (0.14)	0.98 (0.76, 1.27)	
Bronchiolitis	1 (0.01)	0 (0.00)	NE	
Bronchitis	31 (0.22)	77 (0.19)	0.77 (0.49, 1.19)	
Non-suppurative OM	56 (0.11)	43 (0.08)	0.73 (0.48, 1.10)	

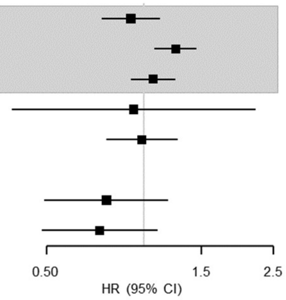

Abbreviations: CI, confidence interval; HR, hazard ratio; NE, not estimable; OM, otitis media; URI, upper respiratory infection.

[a] Results for other adverse drug events are presented in Figure 1 (main manuscript).

© 2022 Butler AM et al. *JAMA Network Open.*

[b] Shaded infections are bacterial infections, others are viral infections.

© 2022 Butler AM et al. *JAMA Network Open.*

eFigure 4. Standardized Mean Differences of Patient- and Provider-Level Characteristics Between Treatment Groups, in the Unweighted and Weighted Populations of Children with Asthma and Allergy or Asthma Exacerbation, for Acute Kidney Failure Safety Outcome Cohort[a,b,c,d]

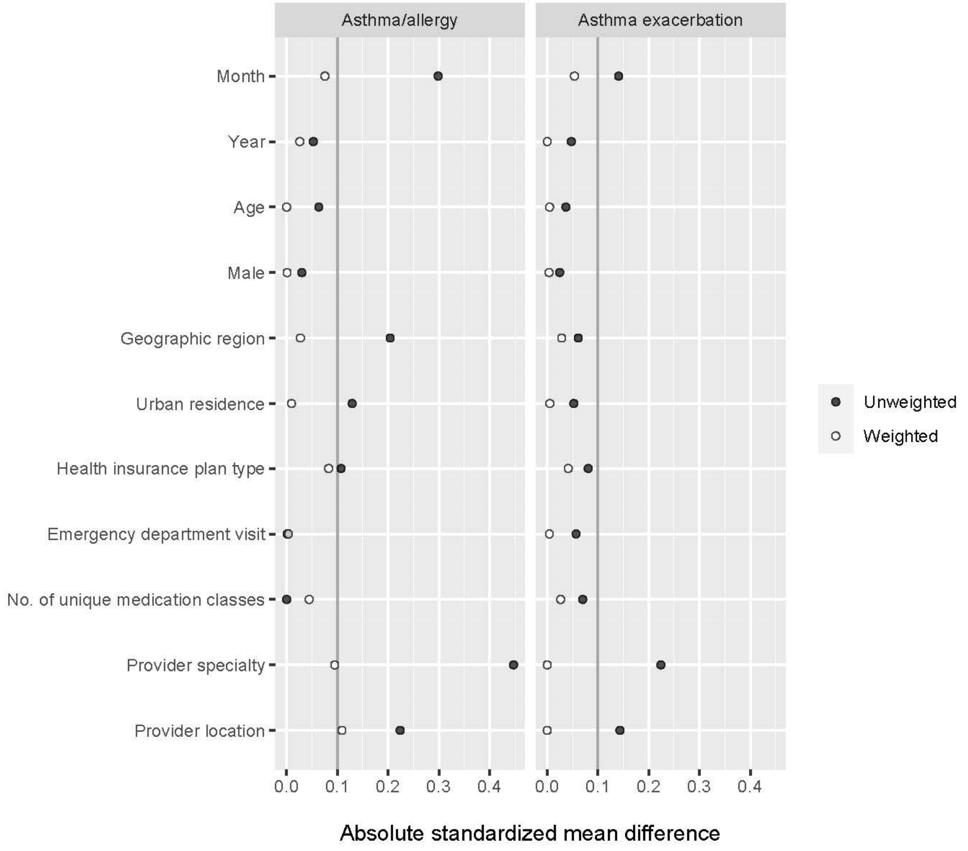

[a] Standardized mean difference (SMD) calculated as the difference in means or proportions of a variable divided by the pooled standard deviation of the variable. All standardized mean difference estimates compare children who received an appropriate antibiotic (reference) versus an inappropriate antibiotic within each infection cohort, after cohort-specific trimming. Standardized mean differences <0.1 indicate no substantial difference in means or proportions between groups. In the inverse probability of treatment (IPT)-weighted population, all measured baseline characteristics were well-balanced between treatment groups (standardized mean differences < 0.10) with the following exceptions: provider location for asthma/allergy for the outcomes of nausea/vomiting/abdominal pain, non-*C. difficile* diarrhea, *C. difficile* infection, anaphylaxis/angioedema/laryngeal edema, skin rash/urticaria, unspecified allergy, and tendinopathy (SMDs 0.11–0.18).

[b] Each outcome was evaluated in a separate cohort with a separate propensity score model and weights. The acute kidney failure cohort is presented here as a representative example because it had few cases of acute kidney failure in the 30 days prior to the index date (i.e., 7 cases in the asthma/allergy cohort) and thus made few exclusions based on previous ascertainment of the outcome.

© 2022 Butler AM et al. *JAMA Network Open.*

[c] Demographics were identified in the month of index. Emergency department visits and unique medication classes were identified in the baseline period before index. Provider specialty and provider location refer to the index diagnosis.
[d] Grey shaded circles reflect overlap of unweighted and weighted SMDs.

© 2022 Butler AM et al. *JAMA Network Open.*

eFigure 5. Propensity Score–Weighted Hazard Ratio Estimates of Adverse Drug Events Following Inappropriate vs Appropriate Antibiotic Prescriptions Among Asthma or Allergy and Asthma Exacerbation Pediatric Cohorts

	No. of events (Rate per 10,000 person-days) Appropriate Agent	Inappropriate Agent	Weighted HR (95% CI)
**Nausea/vomiting/abdominal pain**			
Asthma/allergy	1,935 (3.02)	78 (3.56)	1.09 (0.85, 1.41)
Asthma exacerbation	281 (4.39)	70 (4.12)	0.94 (0.72, 1.22)
**Non-C. difficile diarrhea**			
Asthma/allergy	1,292 (0.96)	50 (1.09)	1.03 (0.74, 1.43)
Asthma exacerbation	187 (1.39)	47 (1.31)	0.96 (0.69, 1.34)
**C. difficile infection**			
Asthma/allergy	6 (0.00)	1 (0.01)	NE
Asthma exacerbation	1 (0.00)	0 (0.00)	NE
**Anaphylaxis/angioedema/laryngeal edema**			
Asthma/allergy	995 (7.06)	15 (3.11)	0.92 (0.46, 1.83)
Asthma exacerbation	23 (1.57)	0 (0.00)	NE
**Skin rash/urticaria**			
Asthma/allergy	1,289 (2.05)	71 (3.26)	1.96 (1.44, 2.66)
Asthma exacerbation	138 (2.13)	57 (3.31)	1.55 (1.13, 2.14)
**Unspecified allergy**			
Asthma/allergy	279 (0.45)	19 (0.88)	2.77 (1.50, 5.11)
Asthma exacerbation	41 (0.63)	11 (0.63)	1.24 (0.57, 2.71)
**Stevens-Johnson syndrome/toxic epidermal necrolysis**			
Asthma/allergy	0 (0.00)	0 (0.00)	NE
Asthma exacerbation	0 (0.00)	0 (0.00)	NE
**Acute kidney failure**			
Asthma/allergy	6 (0.01)	1 (0.05)	NE
Asthma exacerbation	1 (0.02)	0 (0.00)	NE
**Tendinopathy plus tendon rupture**			
Asthma/allergy	787 (0.21)	15 (0.12)	0.45 (0.26, 0.77)
Asthma exacerbation	61 (0.17)	20 (0.20)	1.31 (0.76, 2.24)

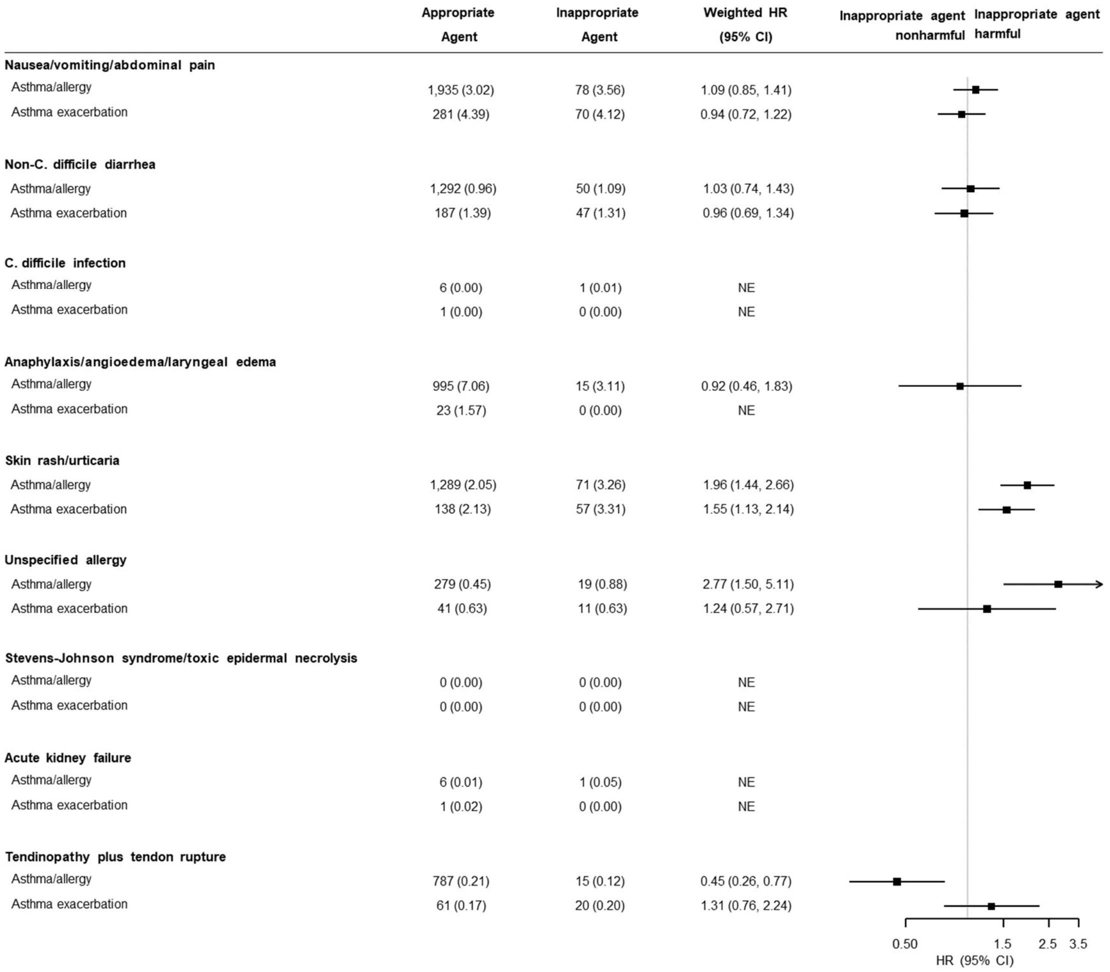

Abbreviations: CI, confidence interval; HR, hazard ratio; NE, not estimable.

© 2022 Butler AM et al. *JAMA Network Open.*

eFigure 6. Weighted 30-Day Attributable Expenditures of Inappropriate Antibiotic Prescriptions for Asthma or Allergy and Asthma Exacerbation Pediatric Cohorts

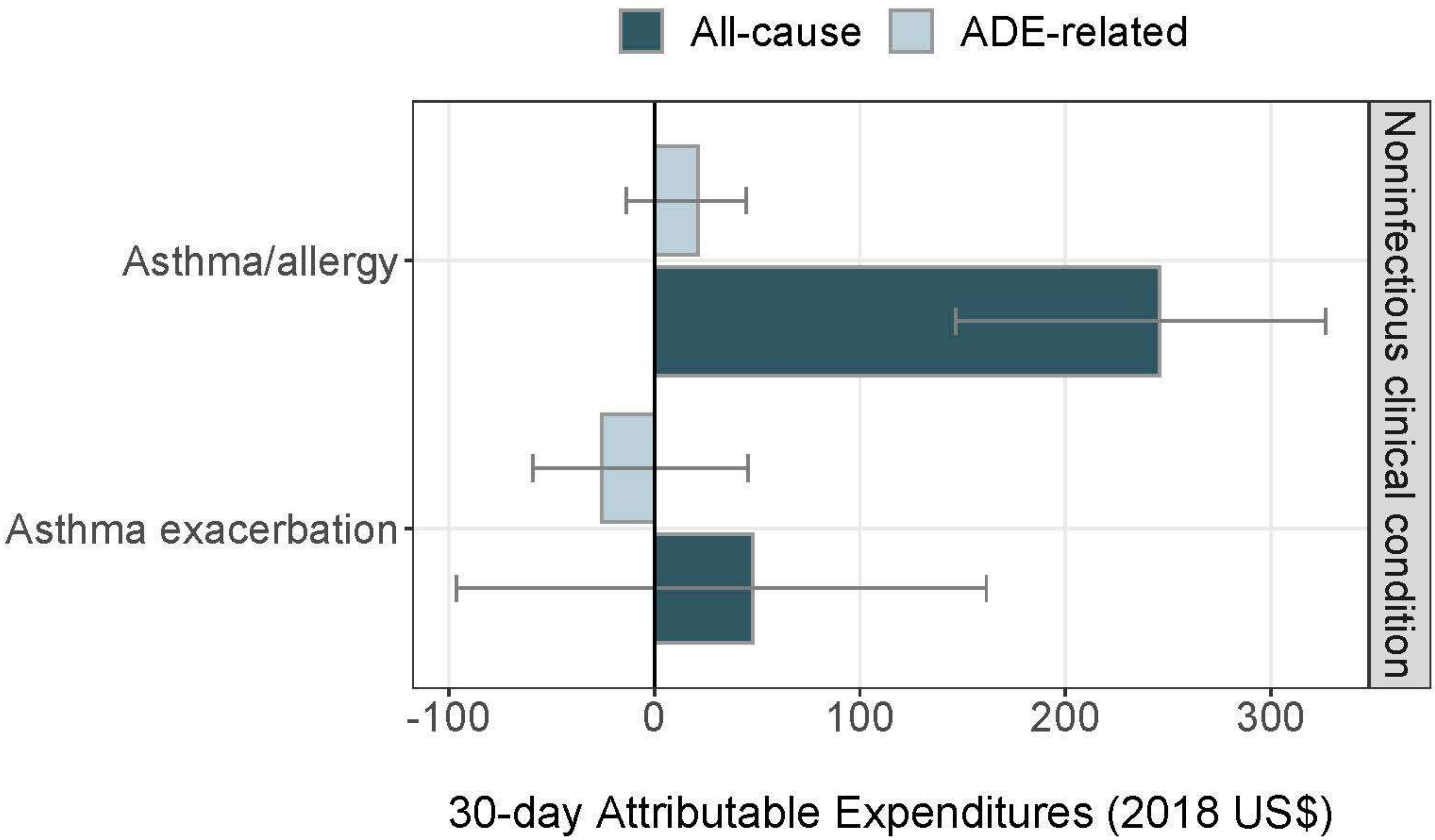

Abbreviations: ADE, adverse drug event.

Grey bars denote 95% confidence interval estimates.

© 2022 Butler AM et al. *JAMA Network Open.*

eReferences.

1. Lieberthal AS, Carroll AE, Chonmaitree T, et al. The diagnosis and management of acute otitis media. *Pediatrics*. Mar 2013;131(3):e964-99. doi:10.1542/peds.2012-3488
2. Shulman ST, Bisno AL, Clegg HW, et al. Clinical practice guideline for the diagnosis and management of group A streptococcal pharyngitis: 2012 update by the Infectious Diseases Society of America. *Clinical infectious diseases : an official publication of the Infectious Diseases Society of America*. Nov 15 2012;55(10):1279-82. doi:10.1093/cid/cis847
3. Chow AW, Benninger MS, Brook I, et al. IDSA clinical practice guideline for acute bacterial rhinosinusitis in children and adults. *Clinical infectious diseases : an official publication of the Infectious Diseases Society of America*. Apr 2012;54(8):e72-e112. doi:10.1093/cid/cir1043
4. Sturmer T, Rothman KJ, Avorn J, Glynn RJ. Treatment effects in the presence of unmeasured confounding: dealing with observations in the tails of the propensity score distribution--a simulation study. *American journal of epidemiology*. Oct 1 2010;172(7):843-54. doi:10.1093/aje/kwq198
5. Stürmer T, Webster-Clark M, Lund JL, et al. Propensity Score Weighting and Trimming Strategies for Reducing Variance and Bias of Treatment Effect Estimates: A Simulation Study. *American journal of epidemiology*. Aug 1 2021;190(8):1659-1670. doi:10.1093/aje/kwab041
6. Austin PC. Balance diagnostics for comparing the distribution of baseline covariates between treatment groups in propensity-score matched samples. *Statistics in medicine*. Nov 10 2009;28(25):3083-107. doi:10.1002/sim.3697

© 2022 Butler AM et al. *JAMA Network Open.*
